# Reprogramming macrophages with R848-loaded artificial protocells to modulate skin and skeletal wound healing

**DOI:** 10.1242/jcs.262202

**Published:** 2024-08-29

**Authors:** Paco López-Cuevas, Tiah C. L. Oates, Qiao Tong, Lucy M. McGowan, Stephen J. Cross, Can Xu, Yu Zhao, Zhuping Yin, Ashley M. Toye, Asme Boussahel, Chrissy L. Hammond, Stephen Mann, Paul Martin

**Affiliations:** ^1^ School of Biochemistry, Biomedical Sciences Building, University Walk, University of Bristol, Bristol BS8 1TD, UK; ^2^ School of Cellular and Molecular Medicine, Biomedical Sciences Building, University Walk, University of Bristol, Bristol BS8 1TD, UK; ^3^ School of Physiology, Pharmacology and Neuroscience, Biomedical Sciences Building, University Walk, University of Bristol, Bristol BS8 1TD, UK; ^4^ Wolfson Bioimaging Facility, Biomedical Sciences Building, University Walk, University of Bristol, Bristol BS8 1TD, UK; ^5^ Centre for Protolife Research, School of Chemistry, University of Bristol, Bristol BS8 1TS, UK; ^6^ National Institute for Health Research Blood and Transplant Research Unit (NIHR BTRU) in Red Blood Cell Products, University of Bristol, Bristol BS34 7QH, UK; ^7^ Max Planck Bristol Centre for Minimal Biology, School of Chemistry, University of Bristol, Bristol BS8 1TS, UK

**Keywords:** Bone, Inflammation, Macrophages, Protocells, Wound healing, Zebrafish

## Abstract

After tissue injury, inflammatory cells are rapidly recruited to the wound where they clear microbes and other debris, and coordinate the behaviour of other cell lineages at the repair site in both positive and negative ways. In this study, we take advantage of the translucency and genetic tractability of zebrafish to evaluate the feasibility of reprogramming innate immune cells *in vivo* with cargo-loaded protocells and investigate how this alters the inflammatory response in the context of skin and skeletal repair. Using live imaging, we show that protocells loaded with R848 cargo (which targets TLR7 and TLR8 signalling), are engulfed by macrophages resulting in their switching to a pro-inflammatory phenotype and altering their regulation of angiogenesis, collagen deposition and re-epithelialization during skin wound healing, as well as dampening osteoblast and osteoclast recruitment and bone mineralization during fracture repair. For infected skin wounds, R848-reprogrammed macrophages exhibited enhanced bactericidal activities leading to improved healing. We replicated our zebrafish studies in cultured human macrophages, and showed that R848-loaded protocells similarly reprogramme human cells, indicating how this strategy might be used to modulate wound inflammation in the clinic.

## Teaser

**Highlighted Article:** Phagocytic uptake of R848-loaded protocells reprogrammes human macrophages *in vitro*, and in zebrafish, models of skin and skeletal repair drives macrophages to be pro-inflammatory and improves healing of infected wounds.

## INTRODUCTION

It is now clear that tumours and wounds share multiple cellular and molecular mechanisms, including a rather similar inflammatory response, which differs only in that it is generally acute and transient in a healing wound but chronic in a cancer scenario (MacCarthy-Morrogh and Martin, 2020; Schäfer and Werner, 2008). Inflammatory cells, in particular macrophages and neutrophils, constitute a major cellular component in the tumour microenvironment (Noy and Pollard, 2014; Powell and Huttenlocher, 2016), and are also some of the first cells to respond to an injury (Weavers and Martin, 2020). In cancer and in wound repair, these innate immune cell lineages have been shown to play both positive and negative roles, thus flagging up the potential benefits that might be offered by the capacity to reprogramme these cells. Already checkpoint inhibitor modulation of the adaptive immune system has proven to provide very powerful anti-cancer therapeutics (Mellman et al., 2011; Sun et al., 2023), and similarly, harnessing the innate immune system has already begun to show powerful anti-cancer effects in several animal models (López-Cuevas et al., 2022; Rodell et al., 2018) and in the clinic (Cassetta and Pollard, 2018). However, innate immunotherapy has been less explored clinically in the context of wound healing, in part due to the lack of treatments that effectively target innate immune cells. Nonetheless, it is clear from zebrafish and mouse studies that the presence of a persistent pro-inflammatory response can dramatically alter wound status (de Kerckhove et al., 2018; López-Cuevas et al., 2021; Raziyeva et al., 2021), and in the case of an infected wound has been shown to speed up and improve the repair process (de Kerckhove et al., 2018).

We have previously shown that miniature artificial protocells can be used as an effective delivery system to target reprogramming cargoes [e.g. anti-microRNAs (miRNAs)] to innate immune cells and enhance their anti-cancer potential in a zebrafish model (López-Cuevas et al., 2022). In this study, we aimed to expand the functionality and application of protocells by loading them with alternative reprogramming cargoes, in this case with the small molecule Resiquimod (R848), to modulate the inflammatory response in the context of several wound scenarios including skin healing and skeletal repair.

R848 is a synthetic agonist of the endolysosomal toll-like receptors 7 and 8 (TLR7 and TLR8) (Hemmi et al., 2002; Jurk et al., 2002), which are known to be primarily expressed in leukocytes and to trigger pro-inflammatory pathways upon ligand binding-mediated activation (Blasius and Beutler, 2010; Wagner et al., 2021). R848 has previously been shown to promote polarization of murine macrophages towards a pro-inflammatory phenotype (Anfray et al., 2021; Epaulard et al., 2014; Rodell et al., 2018; Sallam et al., 2021) and to trigger an upregulation of several pro-inflammatory-related cytokines, including tumor necrosis factor α (TNFα; also known as just TNF), interleukin 1β (IL1β), IL6 and interferon γ (IFNγ), in zebrafish (Bottiglione et al., 2020; Muire et al., 2017; Progatzky et al., 2019; Ward and Martin, 2023).

Because the immunological modulatory activities of R848 are known to be initiated within endosome and lysosome compartments, and our own previous studies have shown that it is mainly macrophages and neutrophils (and to a limited degree also endothelial cells) that engulf protocells and deliver their cargoes precisely to these intracellular compartments (López-Cuevas et al., 2022), we reasoned that the protocell system might be an ideal means to target R848 to TLR7 and TLR8 in macrophages. In this way, we hoped to enhance their pro-inflammatory phenotype, and consequently alter the wound healing process. The potent effect of R848 in different innate immunotherapies against infectious diseases and cancers has been recently reported (Bhagchandani et al., 2021), but it is not known how R848-reprogrammed macrophages might modulate aspects of the wound repair response.

Zebrafish have recently emerged as a good platform to test new synthetic agonists of endosomal TLRs, as recently indicated by several drug development and delivery studies (Ali et al., 2011; MacRae and Peterson, 2015; Rizzo et al., 2013). Comparison of the sequence, cellular expression, ligand specificity and downstream signal transduction pathways of zebrafish and mammalian TLRs have revealed that although some zebrafish TLRs either do not have conserved functions or are specific to zebrafish (Kanwal et al., 2014; Sepulcre et al., 2009), endosomal TLR7, TLR8 and TLR9 are very well conserved (Jault et al., 2004; Kanwal et al., 2014; Li et al., 2017; Meijer et al., 2004; Van Der Vaart et al., 2012; Yeh et al., 2013). This conservation encouraged us to utilize zebrafish as a pre-clinical model to test the potential human applications of endosomal TLR-stimulating drugs, including R848, in the context of wound inflammation.

Here, we specifically target wound-associated macrophages by virtue of their proclivity for phagocytosis, and we deliver pro-inflammatory inducing cargoes (R848) to them through intravenous (IV) injection of protocells. We use the non-lipid microscale proteinosome-based protocells previously reported (López-Cuevas et al., 2022), which are delineated by a semi-permeable membrane consisting of a stabilized, cross-linked monolayer of conjugated protein-polymer building blocks, endowing them with a high stability to changes in temperature, ionic strength, pH and presence of metal ions, and the possibility of encapsulating high levels of guest cargoes, with concentration of encapsulated payload higher than those in bulk solution, which assures sufficient molecule delivery into targeted cells (Huang et al., 2013, 2014; Li et al., 2014; Mukwaya et al., 2020, 2021). None of these advantages are possible in other delivery formulations, such as liposomes.

## RESULTS

### Protocells are taken up by wound macrophages

**Fig. 1.**
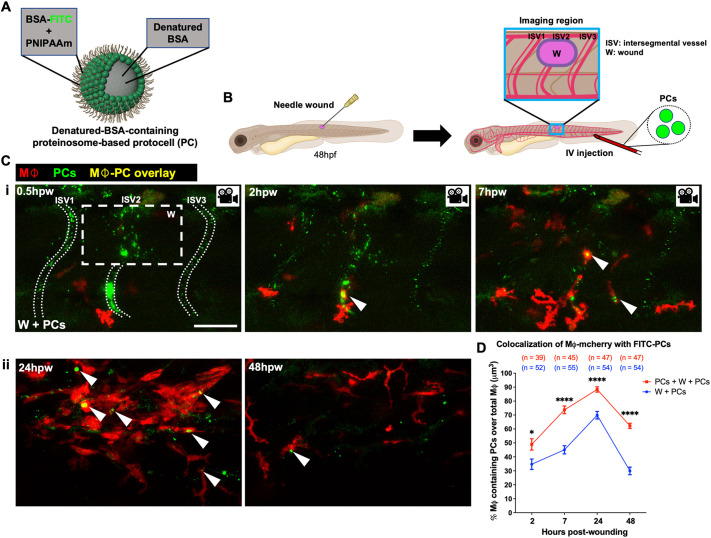
**Protocell engulfment by wound macrophages.** (A) Schematic of denatured-BSA-containing FITC-labelled proteinosome-based protocells (López-Cuevas et al., 2022). (B) Schematic for experiments in C and D showing wounds, injection site and imaging region (blue box). (Ci) Confocal images from a time-lapse movie of a wounded Tg(*mpeg1:mCherry*) larva after FITC-protocell injection, showing protocells at the wound region, and within intersegmental vessels (white dotted lines) or macrophages (white arrowheads) at 0.5, 2 and 7 hpw. (Cii) Confocal images of the same experiment at 24 and 48 hpw. (D) Graph showing percentage of macrophages containing protocell(s) for wounded fish treated with a single (W+PCs) or double (PCs+W+PCs) protocell dose. White dashed box in C indicates wound site; each dot in D represents the mean of all fish analysed. Error bars are mean±s.e.m. *P<0.05; ****P<0.0001 (unpaired two-sided Mann–Whitney test). ISV, intersegmental vessel; Mφ, macrophages; *n*, number of fish; W, wound. Scale bar: 50 μm.

In a previous study, we have shown that miniature artificial proteinosome-based protocells, prepared by the spontaneous assembly and cross-linking of bovine serum albumin-NH_2_ (BSA-NH_2_) and poly(N-isopropylacrylamide) (PNIPAAm) nanoconjugates, are an effective delivery vehicle for anti-miRNA cargoes for reprogramming of macrophages in a cancer microenvironment (López-Cuevas et al., 2022). Here, we wanted to test the use of protocells to modulate macrophage behaviours and activities in a wound repair context. We first investigated whether systemic protocell administration via IV injection might passively deliver protocells to a wound as a consequence of local vascular damage and whether this might lead to uptake of protocells by wound-recruited macrophages. We performed needle-stick flank wounds in 48 h post-fertilization (hpf) zebrafish larvae with fluorescently labelled macrophages [Tg(*mpeg1:mCherry*)] (Ellett et al., 2011) and subsequently IV injected these larvae with a solution of 2 µm-diameter denatured-BSA-containing protocells that had been labelled with fluorescein isothiocyanate (FITC), initially without R848 cargo (Fig. 1A,B). We observed that protocells do indeed leak from the circulation at the wound site, and that wound-recruited macrophages rapidly phagocytose these protocells (Fig. 1C; Fig. S1, Movie 1). Some recruited macrophages already contained protocells prior to their migration towards the wound, which they must have taken up within the circulation (Movie 2). Fluorescently labelled neutrophils in Tg(*lyz:DsRed*) larvae (Hall et al., 2007), also take up protocells at the wound site and were visible alongside other neutrophils already loaded with protocells on their arrival at the wound (Fig. S1, Movie 3).

Live imaging revealed that 70% of macrophages had taken up protocells at 24 h post-wounding (hpw) (Fig. 1C,D). An additional IV delivery of protocells prior to wounding significantly increased the number of wound macrophages with internalized protocells at 2, 7, 24 and 48 hpw (Fig. 1D). This double protocell dosing method administered before and after wounding was used for all our subsequent larval experiments.

### R848-protocells enhance the pro-inflammatory state of wound-associated macrophages and alter their behaviour at the wound site

With the goal of targeting and activating TLR7 and TLR8 receptors in the endosomal compartment of leukocytes, we next loaded protocells with R848 to test whether they could effectively deliver R848 to these leukocytes and whether this might alter their behaviour and phenotype at the wound site.

**Fig. 2.**
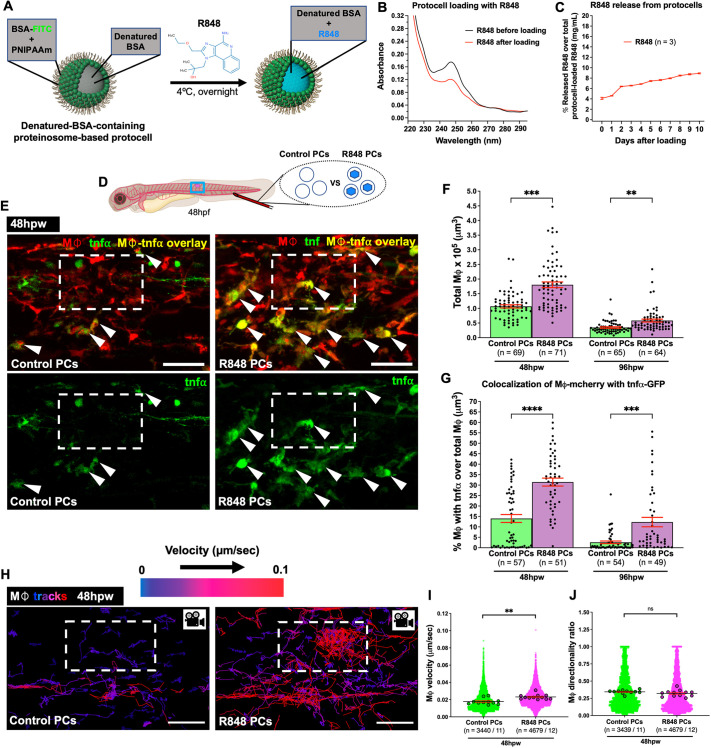
**Uptake of R848-protocells by wound macrophages induces pro-inflammatory reprogramming and alters their behaviour.** (A) Schematic for loading denatured-BSA-containing FITC-protocells with R848. (B) Spectra showing absorbance quantification of the R848 supernatant before and after protocell loading. (C) Graph showing percentage of R848 released from loaded protocells. (D) Schematic for experiments in E–J showing wound, injection site and imaging region (blue box). (E) Confocal images of wounded Tg(*mpeg1:mCherry;tnfα:GFP*) larvae showing TNFα-positive macrophages (white arrowheads) after each protocell treatment. (F,G) Graphs showing total macrophages (F) or percentage of TNFα-positive macrophages (G). Data in G are part of an experiment shown in Fig. S2 that also includes ‘medium’ and ‘free R848’ groups. (H) Confocal images of wounded Tg(*mpeg1:nls-Clover*) larvae showing macrophage tracks in the wound vicinity following each protocell treatment. Warm and cold track colours indicate faster and slower macrophage velocity, respectively. (I,J) Graphs showing velocity (I) or directionality ratio (J) of macrophages. White dashed boxes in E and H indicate wound sites; each dot in C represents the mean of all experiments analyzed, in F and G, one fish, and in I and J, each small dot represents one cell and larger dots are the mean from one fish. Error bars are mean±s.e.m. ns, not significant; **P<0.01; ***P<0.001; ****P<0.0001 (unpaired two-sided Mann–Whitney test). Mφ, macrophages; *n,* number of fish (F,G), number of experiments (C) or number of cells/number of fish (I,J). Scale bars: 50 μm.

We loaded FITC-labelled protocells with R848 by hydrophobic-induced complexation with pre-encapsulated denatured BSA (hereafter, these are denoted R848-protocells) (Fig. 2A). Absorbance data indicate that R848 was successfully loaded into denatured-BSA-containing protocells at a local concentration of ∼7.4 mM (1.3 mM in bulk solution containing 3.2×10^7^ protocells/µl) (Fig. 2B); we found minimal leakage of R848 from the protocells into the external solution at up to 10 days after initial loading (Fig. 2C), indicating a strong and stable bond between R848 and denatured BSA within protocells.

Macrophages play a central role in modulating the overall inflammatory response during wound healing. After tissue injury, wound-recruited macrophages display a pro-inflammatory, or M1, phenotype, which is characterized by the production of reactive oxygen and nitrogen species to kill invading pathogens and the secretion of several pro-inflammatory cytokines (TNFα, IL1β and IL6) and chemokines to amplify the inflammatory response by attracting and activating other immune cells at the wound (Peña and Martin, 2024). These macrophage-related inflammatory events must be tightly regulated as prolonged activation or hyperstimulation can lead to pathological healing, including scarring.

Previous *in vitro* investigations of murine macrophages have demonstrated that R848 exposure leads to increased production of TNFα in these cells (Anfray et al., 2021; Epaulard et al., 2014; Sallam et al., 2021). Therefore, as a phenotypic readout of R848-mediated macrophage polarization we used a TNFα reporter zebrafish line [Tg(*tnfα:GFP*)] (Marjoram et al., 2015) in combination with Tg(*mpeg1:mCherry*) to reveal the presence of TNFα-expressing macrophages. IV injection of R848-loaded protocells in 48 hpf Tg(*mpeg1:mCherry;tnfα:GFP*) larvae resulted in a significantly increased number of total macrophages recruited to the wound and a higher proportion of these wound-associated macrophages expressed the TNFα reporter over the course of 96 hpw than in those fish injected with control protocells (loaded with medium, but no R848) or free R848 drug (Fig. 2D–G; Fig. S2). Similarly, IV injection of R848-protocells in Tg(*mpeg1:mCherry;il1β:GFP*) fish (Ogryzko et al., 2019) revealed an increase in the percentage of IL1β-expressing macrophages at 48 and 96 hpw (Fig. S3). Together, these data suggest that protocells loaded with R848 can enhance both recruitment of macrophages and the pro-inflammatory state of wound macrophages, and that this reprogramming effect seems to be considerably more effective than delivery of the free drug alone.

Control (medium only-injected) larvae displayed comparable levels of TNFα-positive macrophages to those injected with control protocells at 48 and 96 hpw (Fig. S2), indicating that protocells, by themselves, do not trigger a significant inflammatory response.

To investigate how R848-protocell uptake might influence immune cell behaviours in response to tissue damage, we used a bespoke algorithm (López-Cuevas et al., 2021) to automatically track macrophages and measure their speed and directionality as they are drawn to the wound. R848-protocells were IV injected in 48 hpf Tg(*mpeg1:nls-Clover*) larvae (Bernut et al., 2019) in which the macrophage nuclei are labelled to enable automated tracking. Our data showed that wound macrophages that had taken up R848-protocells displayed an altered migratory behaviour, with an increased velocity at 48 hpw (Fig. 2H,I; Movie 4). However, no changes in macrophage directionality ratio were observed between R848-protocell-treated and control fish (Fig. 2J; Movie 4). These results are consistent with previous *in vivo* studies reporting similar behavioural effects in macrophages activated by an infection (López-Cuevas et al., 2021).

### R848-protocell-reprogrammed macrophages lead to increased wound angiogenesis

**Fig. 3.**
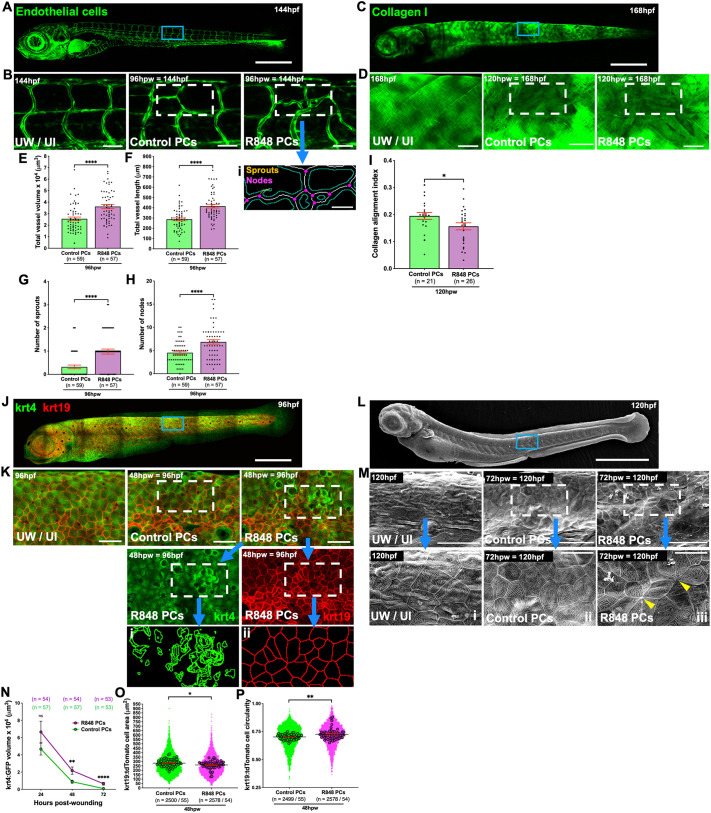
**R848-protocell-mediated reprogramming of wound macrophages delays aseptic wound healing by altering angiogenesis, collagen deposition and re-epithelialization.** (A,C,J,L) Confocal (A,C,J) and SEM (L) images of whole Tg(*fli:eGFP*) (blood vessels) (A), Tg(*krt19:col1α2-GFP*) (collagen) (C), Tg(*krt4:GFP;krt19:tdTomato-CAAX*) (keratinocytes) (J) or WT (L) larvae. (B,D,K,M) High magnification views of unwounded, uninjected (UW, UI) or wounded larvae after each protocell treatment. (Bi,Ki,Kii) Post-software projection images of R848-protocell-treated larvae showing segmentation of endothelial cells (Bi), high-fluorescence krt4 (Ki) and krt19 cells (Kii). (Mi–Miii) High magnification views of M. (E–H) Graphs showing total vessel volume (E) and vessel length (F), and number of vessel sprouts (G) and nodes (H). (I) Graph showing collagen alignment index. (N–P) Graphs showing high-fluorescence krt4–GFP volume (N) and krt19–tdTomato cell area (O) and circularity (P). Blue boxes in A,C,J and L indicate imaging regions; white dashed boxes in B, D, K and M indicate wound sites; yellow arrowheads in M indicate wounded keratinocytes; each dot in E–H and I represents one fish, in N the mean of all fish analysed, and in O and P, each small dot represents one cell and larger dots are the mean from one fish. Error bars are mean±s.e.m. ns, not significant; *P<0.05; **P<0.01; ****P<0.0001 [unpaired two-sided Mann–Whitney test (E–G,N–P) or unpaired two-sided *t*-test (H,I)]. *n*, number of fish (E–H,I,N) or number of cells/number of fish (O,P). Scale bars: 500 μm (A,C,J,L); 50 μm (B,Bi,D,K,M); 20 μm (Mi–iii).

Given that administration of R848-protocells to zebrafish larvae clearly alters the recruitment, behaviour and inflammatory profile of wound-associated macrophages, we next investigated how these reprogrammed macrophages might impact downstream aspects of the wound healing process, for example, wound angiogenesis, which is a consequence of increased demand for oxygen and nutrients in the highly metabolic healing wound (Peña and Martin, 2024). Previous studies have shown that macrophages interact with sprouting blood vessel tips at the wound site and are implicated in driving wound angiogenesis and subsequent vessel regression (Eming et al., 2014; Gurevich et al., 2018). To test how R848-protocell-reprogrammed macrophages might influence wound angiogenesis, we analysed zebrafish expressing a blood vessel reporter [Tg(*fli:GFP*)] (Lawson and Weinstein, 2002) (Fig. 3A) using an automated algorithm to enable vessel segmentation and found that R848-protocell-treated fish exhibited an increase in wound neoangiogenesis at 96 hpw as measured by quantification of individual vessel sprout and node numbers, as well as total vessel volume and length (Fig. 3B,E–H), just as previously observed in fish where macrophages had been activated with lipopolysaccharide (LPS) or IFNγ (Gurevich et al., 2018).

### R848-protocell reprogramming of inflammatory cells leads to alteration in collagen deposition at the wound site

Macrophages have also been shown to be required for directing wound stromal cells to deposit collagen in an aberrant pattern, leading to scarring (Eming et al., 2014; Mori et al., 2008). To examine how our reprogramming of macrophages might alter collagen deposition, we used the Tg(*krt19:col1α2–GFP*) line (Morris et al., 2018), which marks collagen 1 fibrils in the zebrafish skin (Fig. 3C). We analysed wound collagen deposition using an algorithm to quantify alignment index of the collagen 1 fibrils (Morris et al., 2018), and this showed that collagen fibrils in wounds of R848-protocell-treated fish were more randomly aligned and therefore displayed a less orthogonal pattern than in wounds of control-protocell-treated fish where collagen alignment index was significantly higher (Fig. 3D,I).

### Reprogrammed pro-inflammatory macrophages also lead to an increase in wound hyperpigmentation

We and others have previously shown that inflammatory cells drive the recruitment of melanocytes to a wound, resulting in wound hyperpigmentation (Hossain et al., 2021; Lévesque et al., 2013), and that a persistent inflammatory response triggered by bead implantation into the flank muscle of zebrafish larvae causes a more hyperpigmented wound than a transient inflammatory acute wound (Lévesque et al., 2013). Therefore, we assayed the level of pigmentation, as measured by threshold analysis, at the wound site at 48 hpw. We observed hyperpigmented wounds, a feature of ‘chronic’ non-resolving lesions (Passeron et al., 2018; Shah et al., 2012), in wild-type (WT) fish treated with R848-protocells at 48 hpf (Fig. S4), reflecting greater melanocyte recruitment to these wounds compared to those in fish treated with control-protocells.

### R848-protocell-reprogrammed inflammatory cells delay wound re-epithelialization

Zebrafish and mouse studies have previously reported that the persistence of pro-inflammatory macrophages, owing to either infection or anti-miR223 treatment, tends to lead to a delay in wound healing (de Kerckhove et al., 2018; López-Cuevas et al., 2021). To study re-epithelialization of our larval skin wounds after injection with R848-protrocells, we performed confocal microscopy studies on Tg(*krt4:GFP;krt19:tdTomato-CAAX*) fish, which allow imaging of both the superficial (krt4) and basal (krt19) epidermal skin cells (Gong et al., 2002; Morris et al., 2018) (Fig. 3J). Our confocal studies reveal how epithelial defects, characterized by the presence of abnormal, ‘extruded’, high-fluorescence krt4 cells and small and circular krt19 cells, are more apparent in fish where macrophages have been reprogrammed by R848-protocells at 48 hpw (Fig. 3K,N–P). Cell extrusion has been reported to contribute to epithelial remodelling to promote effective wound healing (Iida et al., 2019), and our results are indicative that R848-protocells lead to a delay in this process.

These defects are also clear in scanning electron microscopy (SEM) studies, where we see signs of abnormal re-epithelialization remaining in R848-protocell-treated fish as late as 72 hpw, by which time, control wounds have completely healed and there is no longer any sign of tissue injury (Fig. 3L,M).

### R848-protocell treatment enhances bacterial killing by macrophages and improves healing of infected wounds

To address whether this reprogramming protocol might enhance the bactericidal potential of macrophages, and thus offer potential benefits in an infected wound scenario, we IV injected otherwise WT fish with DsRed-labelled *Escherichia coli* (Fig. S5A). We observed that the bacterial burden was significantly more rapidly cleared over a period of 72 h post-infection (hpinf) in fish co-injected with R848-protocells (Fig. S5B,C). Live imaging studies in Tg(*mpeg1:FRET*) fish at 1 hpinf revealed enhanced microbial uptake by macrophages under this treatment (Fig. S5D–G).

**Fig. 4.**
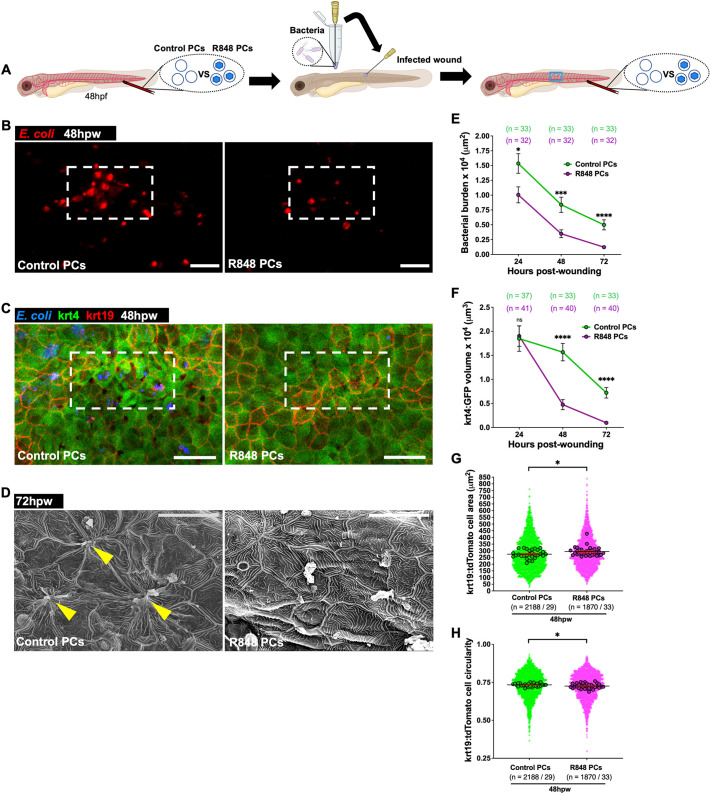
**R848-protocell treatment enhances macrophage bactericidal activities and improves healing of infected wounds.** (A) Schematic for experiments in B–H showing wounds, injection sites and imaging region (blue box). (B) Images of WT larvae with infected wounds showing bacteria (red) after each protocell treatment. (C) Confocal images of infected wounds of Tg(*krt4:GFP;krt19:tdTomato-CAAX*) larvae after each protocell treatment at 48 hpw. (D) SEM images of the same treatments at 72 hpw. (E–H) Graphs showing bacterial burden (E), high-fluorescence krt4–GFP volume (F) and krt19–tdTomato cell area (G) and circularity (H). White dashed boxes in B and C indicate wound sites; yellow arrowheads in D indicate wounded keratinocytes; each dot in E and F represents the mean of all fish analysed, and in G and H, each small dot represents one cell and larger dots are the mean from one fish. Error bars are mean±s.e.m. ns, not significant; *P<0.05; ***P<0.001; ****P<0.0001 (unpaired two-sided Mann–Whitney test). *n*, number of fish (E,F) or number of cells/number of fish (G,H). Scale bars: 100 μm (B), 50 μm (C), 20 μm (D).

We next wanted to investigate whether this protocell strategy might impact the resolution of infected wounds. Fish larvae were locally wounded in the same area as described for sterile wounds above but instead using a needle soaked in DsRed-labelled *E. coli* (Fig. 4A). Infected wounds treated with R848-protocells showed a reduction in bacterial burden at 24, 48 and 72 hpw (Fig. 4A,B,E) and, as a consequence, exhibited much improved wound healing as reflected by a reduction in the number of extruded krt4 cells at 48 and 72 hpw, and by larger and less circular krt19 cells at 48 hpw, compared to what was seen in wounds from control-protocell-treated fish (Fig. 4C,F–H). SEM imaging of R848-protocell-treated wounds showed complete re-epithelialization, whereas we still observed disrupted epithelial skin cells in control infected wounds at 72 hpw (Fig. 4D).

### R848-protocell-mediated macrophage reprogramming leads to altered adult skeletal wound healing

**Fig. 5.**
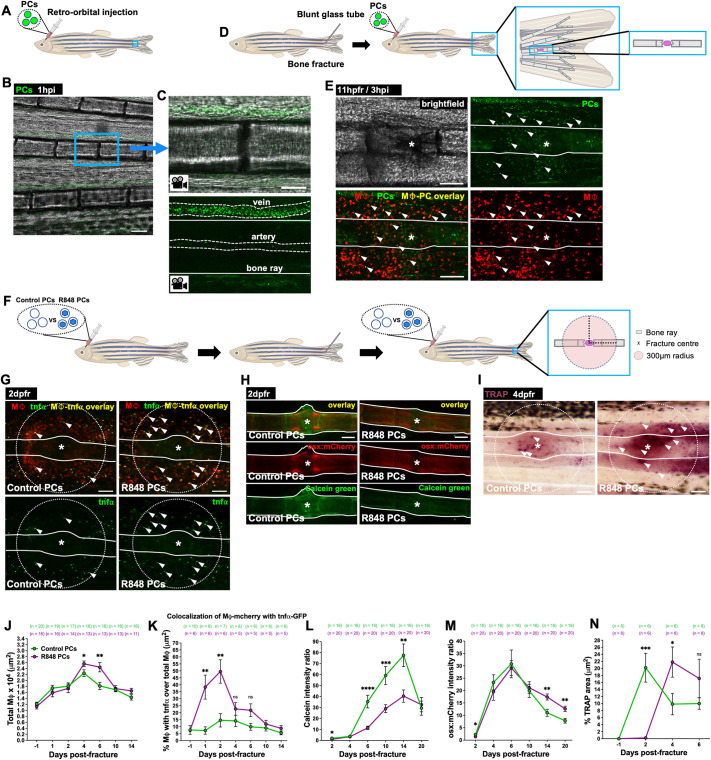
**R848-protocell-mediated macrophage reprogramming impairs bone repair.** (A) Schematic for experiments in B and C showing injection site and imaging region (blue box). (B) Confocal image from a time-lapse movie of an adult caudal fin at 1 h after FITC-protocell injection. (C) High magnification views of B showing FITC-protocells flowing within caudal fin vessels (white dashed lines). (D,F) Schematics for experiments in E and G–N showing fractures, injection sites and imaging regions (blue boxes). (E) Confocal images of a wounded Tg(*mpeg1:mCherry*) adult caudal fin after FITC-protocell injection at 3 h post-injection (hpi), showing protocells within macrophages (white arrowheads). (G–I) Images of fractures in Tg(*mpeg1:mCherry;tnfα:GFP*) (G), Calcein Green-stained Tg(*osx:mCherry*) (H) or TRAP-stained (I) adult caudal fins after each protocell treatment. White arrowheads indicate TNFα-positive macrophages (G) or TRAP-positive regions (I). (J–N) Graphs showing total macrophages (J), percentage of TNFα-positive macrophages (K), and Calcein Green (L) and osx-mCherry (M) fluorescence intensity ratios or percentage of TRAP area (N). White lines in C,E and G–I indicate bone ray margins; white asterisks in E and G–I indicate fracture centre; white dotted circles in G and I indicate analysed regions; each dot in graphs represents the mean of all fish analysed. Error bars are mean±s.e.m. ns, not significant; *P<0.05; **P<0.01; ***P<0.001; ****P<0.0001 [unpaired two-sided *t*-test (J,N) or unpaired two-sided Mann–Whitney test (K–M)]. Mφ, macrophages; *n*, number of fish. Scale bars: 100 μm (B,E,G–I); 50 μm (C).

We wondered whether protocell delivery of pro-inflammatory-inducing cargoes to macrophages might also enable modulation of the inflammatory state of macrophages involved in repair of tissues other than skin, and chose to investigate fracture repair. Fractures in adult fish were made to individual segments of bone in the caudal fin with a blunt-ended glass capillary tube (McGowan et al., 2021) (Fig. 5D). Upon systemic delivery via retro-orbital injection (Pugach et al., 2009) (Fig. 5A–C; Movie 5), FITC-protocells accumulated at the fracture site and were taken up by wound macrophages in Tg(*mpeg1:mCherry*) adult fish (Fig. 5E). When R848-loaded protocells were injected in fracture wounded Tg(*mpeg1:mCherry;tnfα:GFP*) adult fish (Fig. 5F), we observed an elevated number of total wound macrophages and a higher proportion of these cells were expressing the pro-inflammatory TNFα marker just as for skin wounds (Fig. 5G,J,K).

We next investigated whether fin fracture repair was altered after R848-protocell injection. To study bone formation, resorption and remodelling during the bone healing process in adult zebrafish, we analysed the dynamics of osteoblasts, osteoclasts and bone callus (Dietrich et al., 2021). Osteoblasts are the cells responsible for the formation of new bone by secreting bone matrix. This new bone tissue that forms around the fracture site is typically referred as to callus and stabilizes the fracture. Osteoclasts are the cells implicated in degrading bone during the remodelling and bone resorption phase when the callus is reshaped into mature bone. Therefore, in our assays, we first live-stained Tg(*osx:mCherry*) adult fish (with mCherry-tagged osteoblasts) (Singh et al., 2012), with Calcein Green, a fluorescent dye that enables the visualization of newly formed bone by binding to calcium, thus acting as a good indicator of bone callus formation. Injured fish treated with R848-protocells exhibited significantly decreased bone callus formation, as measured by Calcein Green intensity, at 2, 6, 10 and 14 days post fracture (dpfr), and reduced osteoblast recruitment activity at 2 dpfr compared with fish treated with control protocells (Fig. 5H,L,M). We then quantified osteoclast numbers, by staining for tartrate-resistant acid phosphatase (TRAP), an enzyme that is highly expressed in these cells (Burstone, 1959; Minkin, 1982), at the fracture site, and observed that initially there were fewer TRAP-synthesizing osteoclasts at the repair site in R848-protocell-treated fish, but that subsequently, by 4 dpfr, their numbers at the fracture site increased (Fig. 5I,N), mirroring our observations for macrophages in larval skin wounds. Our data show convincingly that macrophage reprogramming can impact repair in tissues outside of skin and might indeed be a novel strategy for also targeting and influencing the inflammatory response at fracture sites.

### Human macrophages are effectively reprogrammed by R848-protocell treatment

**Fig. 6.**
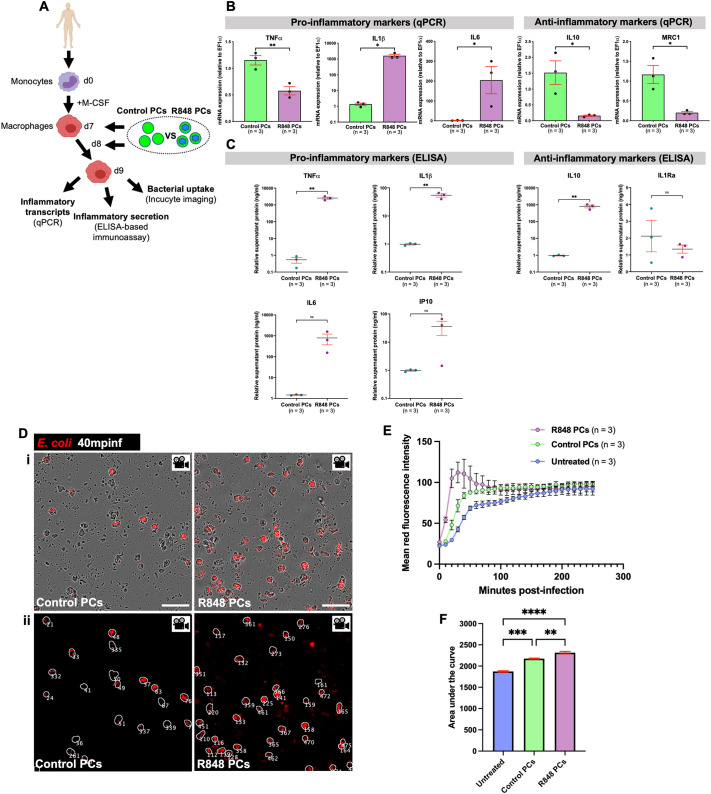
**Uptake of R848-protocells by human macrophages induces a pro-inflammatory state and increases their microbial uptake.** (A) Schematic for experiments in B–F to evaluate R848-protocell-mediated reprogramming of human macrophages. (B) qPCR data of pro- and anti-inflammatory markers in human macrophages after each protocell treatment. (C) Graphs showing quantification of pro- and anti-inflammatory proteins from supernatant of human macrophage cultures after each protocell treatment. (Di) Incucyte images from time-lapse movies showing internalized bacterial particles (red) within human macrophages after each protocell treatment. (Dii) Post-software projection images of Di showing detection of macrophages (white outlines) and number of macrophages (white numbers). (E,F) Graphs showing bacterial load within human macrophages represented as mean fluorescence intensity curves of bacteria inside macrophages (E) and the area under these curves (F). Each dot in B and C represents one experiment, and in E, the mean of all experiments analysed. Error bars are mean±s.e.m. ns, not significant; *P<0.05; **P<0.01; ***P<0.001; ****P<0.0001 [unpaired two-sided *t*-test (B,C) or ordinary one-way ANOVA test with Bonferroni's multiple comparison post-test (F)]. *n*, number of experiments. Scale bars: 50 μm.

To investigate the possibility of using R848-protocells to reprogramme human macrophages with the eventual goal of developing clinically useful therapeutics, we complemented our zebrafish experiments with an *in vitro* human model. Mononuclear cells were isolated from peripheral blood of healthy patients and macrophage differentiation induced with macrophage colony-stimulating factor (M-CSF) (Fig. 6A). To analyse the inflammatory profile of human macrophages following take up of R848-protocells, we utilized quantitative PCR (qPCR) and enzyme-linked immunosorbent assay (ELISA)-based immunoassays as we have previously done after reprogramming macrophages with anti-miR223-loaded protocells (López-Cuevas et al., 2022). Macrophages treated with R848-protocells exhibited an increase in mRNA and secreted protein levels of the pro-inflammatory markers IL1β and IL6, an increase in another pro-inflammatory marker, interferon γ-induced protein 10 (IP10), at the protein level, and a decrease in the anti-inflammatory markers IL10 and mannose receptor c-type 1 (MRC1), and interleukin-1 receptor antagonist (IL1Ra), at the mRNA level and protein level, respectively (Fig. 6B,C). A significant upregulation of TNFα was only detected at the protein level in macrophages supplemented with R848-protocells (Fig. 6C), in agreement with previous *in vitro* studies (Asami et al., 2013; Epaulard et al., 2014; Sallam et al., 2021). Some of the discrepancies we observed between mRNA and protein levels for different inflammatory markers might be explained by protein translation delay, and future time-course experiments will be needed to monitor temporal expression dynamics (Liu et al., 2016).

Next, we wanted to determine whether these reprogrammed pro-inflammatory macrophages had an enhanced bacterial uptake as with our *in vivo* zebrafish studies. To address this, we exposed R848-protocell-treated human macrophages to DsRed-labelled *E. coli* bioparticles. Live Incucyte imaging revealed an increase in bacterial uptake by macrophages under this treatment, with a peak in bacterial load at 40 min post infection (mpinf) (Fig. 6D–F; Movie 6). These data indicate that this R848-protocell approach is indeed an effective reprogramming method for human cells and thus has potential for translational application.

## DISCUSSION

Reprogramming innate immune cells for potential clinical benefit is an aspiration extending back decades (Coley, 1910; Felgner et al., 2016; Wiemann and Starnes, 1994). In recent years, most efforts have been focused on the use of this immunotherapy strategy for anti-cancer applications, leading to the development of successful treatments against several cancers (Mellman et al., 2011; Sun et al., 2023). This suggests that harnessing the immune system is feasible and can lead to positive clinical outcomes. However, innate immune cell modulation for altering wound status is a considerably less explored field in the clinic. Recent mouse studies have demonstrated that activation of the wound inflammatory response can improve tissue repair of infected wounds (de Kerckhove et al., 2018). Despite these promising findings, we are still in need of testing more therapeutically viable systems to enhance innate immune cell activity in a wound context.

Agonist drugs targeting the endolysosomal TLR7, TLR8 and TLR9 have shown remarkable reprogramming effects in immune cells, switching them towards a more pro-inflammatory state. One example of this is R848, a dual TLR7 and TLR8 synthetic agonist previously shown to promote an anti-cancer and anti-bacterial immune response (Bhagchandani et al., 2021), suggesting that it might also have relevance in wound healing settings as well. To investigate the potential of R848 to reprogramme wound inflammation and subsequently influence tissue repair more effectively than by simply delivering free R848, we took advantage of our previously established protocell delivery system (López-Cuevas et al., 2022) to target the R848 drug to macrophages. We chose protocells over other potential delivery vectors because they offer high stability, extensive circulation times and low toxicity *in vivo*. They enable encapsulation and delivery of high concentrations of the reprogramming cargoes, and they also have a high uptake by macrophages (López-Cuevas et al., 2022).

In this study, we exploited the translucency of zebrafish to test the feasibility of reprogramming wound-associated macrophages with R848-loaded protocells. We performed high resolution *in vivo* imaging of fluorescently labelled protocells and showed that protocells injected intravenously accumulate at the wound site and are taken up by wound-associated macrophages in zebrafish. When protocells were loaded with R848 cargoes, macrophages changed their behaviour and interactions with the wound, and switched to a pro-inflammatory phenotype following protocell uptake. This R848-protocell-mediated macrophage reprogramming led to changes in wound angiogenesis and collagen deposition, which in turn, impacted tissue repair, affecting re-epithelialization of the healing wound. Although healing of aseptic skin wounds was somewhat impaired by R848-protocell delivery, this treatment had an opposite effect in infected wounds, improving tissue repair. We upscaled the R848-protocell-mediated reprogramming of macrophages in adult zebrafish in the context of more clinically relevant wounds, including bone fracture, and observed altered osteoblast and osteoclast recruitment, also affecting bone mineralization and skeletal wound repair.

Although more direct evidence will be necessary to show the specificity of the interactions of R848 with TLR7 and TLR8 in zebrafish, sequence comparison and functional studies have demonstrated that the R848 targets, TLR7 and TLR8, are highly conserved between zebrafish and humans (Bottiglione et al., 2020; Jault et al., 2004; Meijer et al., 2004; Progatzky et al., 2019), suggesting that our findings with R848-protocells in zebrafish might be extrapolated into the clinic. In this regard, we showed that human macrophages *in vitro* can also be reprogrammed by R848-protocells and mimic some of the beneficial wound healing behaviours observed in zebrafish, further supporting how this strategy might have clinical applications for treating human wounds.

Moreover, zebrafish could also be a good system to test whether protocell delivery of similar agonists targeting endosomal TLRs, such as imiquimod and 852A (TLR7 agonists), 3M-052 (a dual TLR7 and TLR8 agonist) and IMO-2055 (a TLR9 agonist), which have previously shown to activate murine leukocytes (Cassetta and Pollard, 2018), might be ‘repurposed’ to induce an effective macrophage reprogramming in a clinical wound context.

In summary, our studies show the feasibility of reprogramming innate immune cells at a wound site following tissue damage to the skin or skeleton. Miniature artificial protocells are an efficient means for specifically targeting reprogramming cargoes to immune cells *in vivo*, and we show how activation of endosomal TLRs in this way can effectively change macrophage behaviour at the wound site, maintaining a pro-inflammatory state and improving microbicidal activity which is crucial for some chronic skin wounds and for prevention of osteomyelitis following joint replacement and other skeletal surgeries. Here, and in previous studies, our cargoes have been selected to maintain a pro-inflammatory phenotype in recruited inflammatory cells, but clearly reprogramming in other ways might be more therapeutically beneficial in some clinical scenarios. It will also be possible to refine delivery better by bespoke surface engineering of protocells, for example, by addition of tissue targeting receptors, to enhance the capacity for them to home to wound sites. In the longer term, we envisage protocell delivery becoming a mainstream strategy for clinical scenarios requiring management of the inflammatory response including tissue repair and cancer, as well as for other related inflammatory conditions.

## MATERIALS AND METHODS

### Preparation of proteinosome-based protocells

Denatured BSA (Sigma) was prepared by dissolving BSA in H_2_O (20 mg/ml) and then heating in oil bath (100°C) for 2 h.

Proteinosome-based protocells (unlabelled or FITC labelled) were prepared by mixing an aqueous BSA-NH_2_ and PNIPAAm, or BSA-NH_2_–FITC and PNIPAAm solution with denatured BSA, O,O′-bis[2-(N-succinimidyl-succinylamino)ethyl]polyethylene glycol and 2-ethyl-1-hexanol, followed by sonication, as previously described (López-Cuevas et al., 2022). The upper oil layer of this solution containing cross-linked protocells was collected at 48 h post sedimentation, centrifuged (11,600 ***g***, 10 min), washed and dispersed in H_2_O, and then preserved at 4°C. Protocells numbers were quantified as previously described (López-Cuevas et al., 2022).

### R848 loading into protocells

For R848 (Sigma) loading into protocells, the solution containing denatured-BSA-containing protocells (50 μl, 1.25×10^7^ protocells/μl) was centrifuged (11,600 ***g***, 10 min) and incubated with R848 solution (20 μl, 3.2 mM) at 4°C (overnight). The following day, the mixture was centrifugated (11,600 ***g***, 10 min), washed and then dispersed in H_2_O (20 μl, 3.2×10^7^ protocells/μl, 1.3 mM).

The R848 loading efficiencies into protocells were monitored by UV–visible absorption spectra. In particular, the R848 concentration in protocells was determined by measuring the absorbance (at 247 nm) of R848 solution in supernatant before and after loading into protocells.

### Preparation of bacteria

For fish infection experiments, *E. coli* (BL21) inoculum expressing DsRed (kindly provided by Will Wood, University of Edinburgh, UK) or blue fluorescent protein (BFP) (Thermo Fisher Scientific) was prepared from an overnight LB culture in the log-phase of growth resuspended in 2% polyvinylpyrrolidone 40 (PVP40) solution (Sigma) in PBS with Phenol Red (Sigma). PVP40 was used to improve homogeneity of bacterial suspension, preventing bacteria from sinking and sticking to the inner wall of the injection needle and therefore increasing reproducibility of injecting inoculum during experiments. Phenol Red helped the visualization of the injection process (Benard et al., 2012).

### PBMNC and CD14^+^ isolation

Peripheral blood mononuclear cells (PBMNCs) were isolated from platelet apheresis blood waste (NHSBT, Bristol, UK) from anonymous healthy donors. PBMNC separation was performed using PBMNC Spin Medium (pluriSelect Life Science) as previously described (Bell et al., 2013; van den Akker et al., 2010). Briefly, blood from apheresis cones was diluted 1:1 with Hank's Balanced Salt Solution (Sigma) containing 0.6% acid citrate dextrose and layered over PBMNC spin medium. Samples were centrifuged to generate a density gradient and PBMNCs collected from the interface. CD14^+^ cells were isolated from PBMNCs using a magnetic micro-bead CD14^+^ kit (Miltenyi Biotec), LS columns (Miltenyi Biotec), and MidiMACS separators (Miltenyi Biotec) as previously described (Heideveld et al., 2018).

### Human macrophage *ex vivo* culture, and protocell and bacteria administration

Cells were resuspended at a density of 0.17×10^6^–0.33×10^6^ cells/ml in RPMI 1640 (Gibco) supplemented with 10% FBS, 25 ng/ml M-CSF (Miltenyi Biotec), and penicillin-streptomycin (Sigma) at 100 and 0.1 U/mg per ml of medium, respectively. Cells were incubated at 37°C with 5% CO_2_. Cells were harvested from the adherent macrophage culture via scraping. 4×10^5^ macrophages were used for transcript and protein analyses per treatment for each experiment. Macrophages from different donors were used for each experiment. In all *in vitro* experiments, protocells were administered to macrophage cultures in a high concentration (macrophage:protocell ratio=1:100), and in multiple doses (days 7 and 8), as previously established (López-Cuevas et al., 2022), and macrophages were harvested on day 9 for analysis.

In bacterial uptake assays, pHrodo™ BioParticles™ (Invitrogen) were added to macrophages as per the manufacturer's instructions on day 9 and immediately imaged utilizing the Incucyte imaging system.

### RNA extraction, cDNA synthesis and qPCR

Total RNA from cultured human macrophages was extracted with the TRIzol-Chloroform method (Qiagen). Maxima first strand cDNA synthesis kit (Thermo Fisher Scientific) was used to synthesize cDNA from extracted mRNAs. qPCR was performed with PowerUp SYBR green master mix (Thermo Fisher Scientific) and run in a QuantStudio 3 real-time PCR system. Data from qPCR were normalized to elongation factor 1 α (EF1α). Primer sequences used for mRNA amplification are listed in Table S1.

### ELISA-based multiplex immunoassay for analysis of protein secretion by human macrophages

Supernatant protein levels for human IL1β, IL6, TNFα, IP10, IL10 and IL1Ra were measured using a LEGENDplex Human M1/M2 Macrophage Panel kit (Biolegend) following the manufacturer's instructions. Supernatants from cultured human macrophages were collected in a tube and centrifuged (19,000 ***g***, 10 min) at 4°C to remove particulates, the clarified medium was transferred into a clean tube and undiluted samples were added to a 96-well plate as per the manufacturer's instructions. Samples were acquired using a MACSQuant flow cytometer (Miltenyi Biotec) and analysed using FlowJo Version 10.7 (BD Biosciences).

### Zebrafish lines and maintenance

Husbandry of adult zebrafish (*Danio rerio*) was performed as previously described (Westerfield, 2007). WT and transgenic lines including Tg(*mpeg1:mCherry*)gl23 [referred to as Tg(*mpeg1:mCherry*)] (Ellett et al., 2011), Tg(*lysC:DsRed2*)nz50 [referred to as Tg(*lyz:DsRed*)] (Hall et al., 2007), Tg(*mpeg1.1:CFP-DEVD-YFP*)sh266 [referred to as Tg(*mpeg1:FRET*)] (kind gift from Nikolay Ogryzko and Stephen Renshaw, University of Sheffield, UK), Tg(*kdrl:mCherry-CAAX*)y171 [referred to as Tg(*kdrl:mCherry-CAAX*)] (Fujita et al., 2011), Tg(*mpeg1.1:NLS-mClover*)sh436 [referred to as Tg(*mpeg1:nls-Clover*)] (Bernut et al., 2019), Tg(*fli1:EGFP*)y1 [referred to as Tg(*fli:GFP*)] (Lawson and Weinstein, 2002), TgBAC(*tnfα:GFP*)pd1028 [referred to as Tg(*tnfα:GFP*)] (Marjoram et al., 2015), TgBAC(*il1β:eGFP*)sh445 [referred to as Tg(*il1β:GFP*)] (Ogryzko et al., 2019), Tg(*Ola.Sp7:mCherry*)pd43 [referred to as Tg(*osx:mCherry*)] (Singh et al., 2012), Tg(*krt1-19e:col1a2-EGFP*)zf2175 [referred to as Tg(*krt19:col1α2–GFP*)] (Morris et al., 2018), Tg(*krt4:GFP*)gz7 [referred to as Tg(*krt4:GFP*)] (Gong et al., 2002) and Tg(*krt1-19e:Tomato-CAAX*)zf2176 [referred to as Tg(*krt19:tdTomato-CAAX*)] (Morris et al., 2018) were maintained on a Tüpfel long fin (TL), Ekkwill (EKK) WT or casper background (White et al., 2008).

### Animal and primary human cell ethics statement

The zebrafish studies were reviewed and approved by the University of Bristol Animal Welfare and Ethical Review Body (AWERB), and were carried out under UK HO license number PPL PP3332518. For the human primary cell work, PBMNCs were isolated from platelet apheresis blood waste (NHSBT, Bristol, UK) from anonymous healthy donors with informed consent. Ethics approval for all experimental protocols with these cells was granted by Bristol Research Ethics Committee (REC 12/SW/0199), and methods were carried out in accordance with approved guidelines.

### Protocell and bacteria injection in zebrafish larvae and adults

Larvae were anaesthetized at 48 hpf using 0.16 mg/ml tricaine (Sigma MS-222) and injected systemically with a 2 nl volume containing a protocell solution at a high concentration (1.25×10^7^ protocells/µl, previously reported to be the maximum level that is well tolerated by zebrafish larvae; López-Cuevas et al., 2022), a bacterial solution (400–500 colony-forming units), free R848 or medium alone into the caudal vein using a glass needle (Harvard Apparatus), as previously described (Takaki et al., 2013).

Young adult fish (6 months old) were anaesthetized in 0.16 mg/ml tricaine (Sigma MS-222) and injected systemically, via retro-orbital injection, with protocells (4 µl/fish) using a Hamilton syringe as previously described (Pugach et al., 2009).

Wounded larvae or adults received a dose of protocells immediately after or 8 h post-wounding, respectively, and where stated, an additional protocell injection was given 4 h or 24 h before wounding in larvae or adults, respectively.

In all larval and adult experiments, R848 (free or loaded into protocells) was used at 0.5 mM (in bulk solution containing 1.25×10^7^ protocells/µl).

### Wounding protocols

For larval skin wound experiments, fish were anaesthetized at 48 hpf in 0.16 mg/ml tricaine (Sigma MS-222) prior to wounding their flanks in the dorsal somite opposite the cloaca with a 30-gauge needle (Becton Dickinson), as previously described (Gurevich et al., 2018). For infected skin wound experiments, fish were wounded as for aseptic wounds but now for each larva the point of the 30-gauge needle was dipped in a tube containing a 20 μl *E. coli* pellet.

For adult fin fracture, 6-month-old fish were anaesthetized in 0.16 mg/ml tricaine (Sigma MS-222) and subsequently individual segments of their caudal fin bones were fractured using a blunt-ended glass capillary tube (Harvard Apparatus), as previously described (McGowan et al., 2021). Fractures were induced proximal to the fish body before the first bifurcation in the bone ray (McGowan et al., 2021).

### Stainings of adult zebrafish fins

Calcein Green stain was composed of 40 μM Calcein Green powder (Sigma) dissolved in aquarium system water and pH adjusted to 8 using 1 M NaOH. Live fish were immersed in Calcein Green solution (45 min), and then washed before imaging.

A colorimetric TRAP staining protocol to detect osteoclast activity was performed as previously described (Ethiraj et al., 2022). Fins were amputated, fixed in 4% paraformaldehyde (PFA) (40 min, room temperature) and washed with PBS with 0.1% Triton X-100 (0.1% PBSTX) three times for 5 min each time. Fins were then incubated in TRAP solution (3 h, room temperature), washed in PBS with 0.1% Tween 20 (0.1% PBSTW) three times for 5 min each time and then re-fixed in 4% PFA (30 min, room temperature, in dark). Samples were washed in 0.1% PBSTW three times for 5 min each time, bleached with 0.5% KOH and 3% H_2_O_2_ (30 min) for pigmentation removal, washed again and stored in 90% glycerol at 4°C until imaged with a Leica MZ10 F Stereomicroscope.

### Live imaging of zebrafish and human macrophages

Anaesthetized larvae were mounted in 1% low-melting agarose (Sigma) in glass-bottomed dishes with Danieau's solution and 0.16 mg/ml tricaine (Sigma MS-222), and imaged using a Leica TCS SP8 AOBS confocal laser scanning microscope with 20× glycerol lens or 25× water-dipping lens, at 28°C. Images were processed using Fiji software, and displayed as maximum projections. Images of whole larvae were processed using Fiji and constructed by ‘tiling’ maximum projection of several micrographs together.

Anaesthetized adults were placed in dishes with system H_2_O and 0.16 mg/ml tricaine (Sigma MS-222), and imaged using a Leica MZ10 F Stereomicroscope.

Human macrophages on day 9 of culture were harvested via cell scraping and plated in a 24-well plate at 2×10^5^ cells per well in RPMI 1640 supplemented with 10% FBS, 25 ng/ml M-CSF and penicillin-streptomycin at 100 and 0.1 U/mg per ml of medium, respectively, and placed in the Incucyte imaging system.

### Scanning electron microscopy

Larvae were fixed in primary fix (2.5% glutaraldehyde, 4% PFA and 0.1 M sodium cacodylate) at 4°C overnight. Samples were washed in 0.1 M sodium cacodylate (3×10 min, room temperature) and then incubated in secondary fix (1% osmium tetroxide, 0.1 M sodium cacodylate) at room temperature for 3 h. After fixation, samples were rinsed and serially dehydrated in ethanol, critically point dried and sputter coated with gold and palladium prior to imaging with a FEI Quanta 200 FEG scanning electron microscope.

### Post-image analysis

All image analysis was performed in Fiji (Schindelin et al., 2012). Detection, tracking and spatial analysis of cells or protocells used Modular Image Analysis (MIA) automated workflow plugin for Fiji (Cross et al., 2023). The numerical values for the settings were derived empirically and chosen to accurately represent the fluorescence signal. Post-image quantifications (Q) were performed as below.

#### Q1 – protocell and bacteria uptake by macrophages

The volume of protocells or bacteria inside or outside fish macrophages were automatically quantified from *z*-stack images using the following method. Raw images were optionally passed through a 2D median filter to remove noise and binarized with an Otsu threshold (Otsu, 1979). Prior to threshold application, the calculated threshold was systematically adjusted with a user-controlled multiplication factor and subject to a minimum permitted threshold to prevent segmentation of background. Objects were identified as contiguous foreground-labelled regions (Legland et al., 2016). Detected objects smaller than a user-defined threshold volume were removed from further analysis.

#### Q2 – total macrophages and bacterial burden

The number of macrophages and bacteria were automatically quantified from *z*-stack or single-slice images. Macrophages were detected using a similar method as described in ‘Q1’, except with a Gaussian filter to reduce noise prior to thresholding.

#### Q3 – TNFα- and IL1β-expressing macrophages

The volume of TNFα or IL1β inside or outside macrophages was automatically quantified from *z*-stack images using the same method as described in ‘Q1’. In bone fractures, a 300 μm-radius circular region predefined by the user having the fracture centrally positioned was automatically drawn and analysed. This workflow also offered users the option of manually removing non-macrophage objects that had been accidentally detected.

#### Q4 – macrophage behaviour

Macrophage nuclei were automatically detected from *z*-stack images using a thresholding-based approach. Images were first processed with a Gaussian filter and rolling ball background subtraction, followed by binarization using the moments autothresholding algorithm (Tsai, 1985). An intensity-based watershed transform was used to separate adjacent objects which had become merged during binarization. Objects were detected from the optimized binary image using connected components labelling (Legland et al., 2016). As well as detecting macrophage nuclei, the algorithm tracked macrophage nuclei between frames in time-lapse movies using the TrackMate plugin for Fiji (Tinevez et al., 2017). Behavioural features, including velocity and directionality ratio, were measured for each tracked cell. Directionality ratio values ranged between 0 and 1, with the most directed or persistent movements having a value closer to 1.

#### Q5 – vessels

Branching in blood vessels was measured in *z*-stack images. To enhance blood vessel contrast and reduce gaps in fluorescence, a custom UNet pixel classification model was applied using DeepImageJ (Gómez-de-Mariscal et al., 2021; Ronneberger et al., 2015). The resulting probability image was binarized at a probability of 0.5 and the binary image optimized using successive 3D dilate, erode and fill holes steps. Vessel fragments larger than 100 µm^3^ were identified as objects using connected components labelling (Legland et al., 2016). The segmented vessel objects were skeletonized using the Analyze Skeleton ImageJ plugin (Arganda-Carreras et al., 2010) and edges meeting the following criteria were classified as ‘sprouts’: (1) a single connection to the skeleton, (2) not resulting from clipping of the vessel at the image edge and (3) shorter than a user-specified length.

#### Q6 – collagen

Analysis of collagen was performed as previously described (Morris et al., 2018). Collagen fibril orientation was automatically analysed from *z*-stack images using the ‘Directionality’ plugin for ImageJ with amendments to split the results into angle ranges of 0 to 90 and 0 to −90 degrees. Additionally, the alignment index (AI; equation below) was measured for the two aforementioned angle ranges (Sun et al., 2015). 

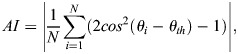
where *θ* is an angular measurement, *θ*_*th*_ is the mean orientation angle and *N* is the total number of angular measurements. An alignment of 1 would indicate no angular dispersion and therefore full fibril alignment whereas a value of 0 would reflect fibrils that are randomly aligned. Prior to alignment calculation, background was subtracted from the angular histogram to improve the signal-to-noise ratio.

#### Q7 – krt4 superficial and krt19 basal skin cells

krt4 volume and krt19 cell area and circularity were automatically quantified from confocal images. Cell segmentation was achieved using the Cellpose deep learning algorithm with the built-in ‘cyto’ model applied to a maximum intensity projection of the red fluorescent channel corresponding to krt19 (Stringer et al., 2020). For detected cells, area and circularity were measured in a user-defined wound location. Regions of the *z*-stack which were positive for the cytoplasmic krt4 signal were identified using intensity-based binarization, with the applied global threshold value set relative to the mean intensity of a user-defined reference region (selected away from the wound). In wounded samples, the volume of identified cytoplasm inside the previously segmented wound was measured.

#### Q8 – bacteria uptake by human macrophages

Bacteria inside or outside human macrophages were automatically quantified from time-lapse fluorescence images. The green fluorescence channel was used for cell detection; this channel was first processed with rolling ball background subtraction (radius=50 px) followed by intensity normalization to assist with detection consistency. Cells were detected from the optimized green image using Cellpose with the built-in ‘cyto’ model (Stringer et al., 2020), with any smaller than a user-defined threshold discarded from further analysis. For all detected cells, the coincident red channel intensity from bacteria was measured as a function of time.

#### Q9 – pigmentation

Pigmentation was automatically quantified on the brightfield channel of *z*-stack images by applying a Gaussian-based stack focuser and then a binary image of the pigment pattern was created, enabling quantification of the percentage area that contained pigment in a region of interest.

#### Q10 – osx:mCherry and Calcein Green

The fluorescence intensity ratios for osx:mCherry and Calcein Green in fractures from single-slice images were measured within a region of interest in Fiji software by dividing the average intensity for each fracture by the average intensity of uninjured bone in the same fish.

#### Q11 – TRAP

The positive area for TRAP staining was automatically quantified in fractures from single-slice images using the same method as described in ‘Q9’ and in a pre-defined user region as per ‘Q3’.

The software used in this study for post-image analysis with Fiji, the MIA plugin, and the corresponding workflows for quantification, unless otherwise referenced, were developed by S.J.C. and can be found at https://zenodo.org/records/10808914.

### Statistical analysis

Statistical analyses and graph design were performed using GraphPad Prism 9 (Version 9.1.0). Data were confirmed to be normally distributed via D'Agostino–Pearson omnibus or Shapiro–Wilk tests prior to further comparisons. When the data were normally distributed, an unpaired two-sided *t*-test or ordinary one-way ANOVA test with Bonferroni's multiple comparison post-test were used to compare two groups or more than two groups, respectively. For non-normally distributed data, an unpaired two-sided Mann–Whitney test or Kruskal–Wallis test with Dunn's multiple comparison post-test were used for comparison between two groups or more than two groups, respectively. Statistical significance is indicated on graphs using standard conventions, as follows: non-significant (ns), *P*≥0.05, *P<0.05, **P<0.01, ***P<0.001, ****P<0.0001. Sample size (*n*), including number of fish, used in the experiments are indicated on graphs. All graphs display mean±s.e.m. where appropriate. All data represented in graphs are pooled from three independent experiments, except for Fig. 2B where data are from one experiment.

## Supplementary Material



10.1242/joces.262202_sup1 Supplementary information
