# Supplementary material for: Reprogramming macrophages with R848-loaded artificial protocells to modulate skin and skeletal wound healing
Source: J Cell Sci. 2024 Aug 29;137(16):jcs262202. doi: 10.1242/jcs.262202 (PMC11385641; doi:10.1242/jcs.262202)
Supplement: Supplementary information [file joces-137-262202-s1.pdf]

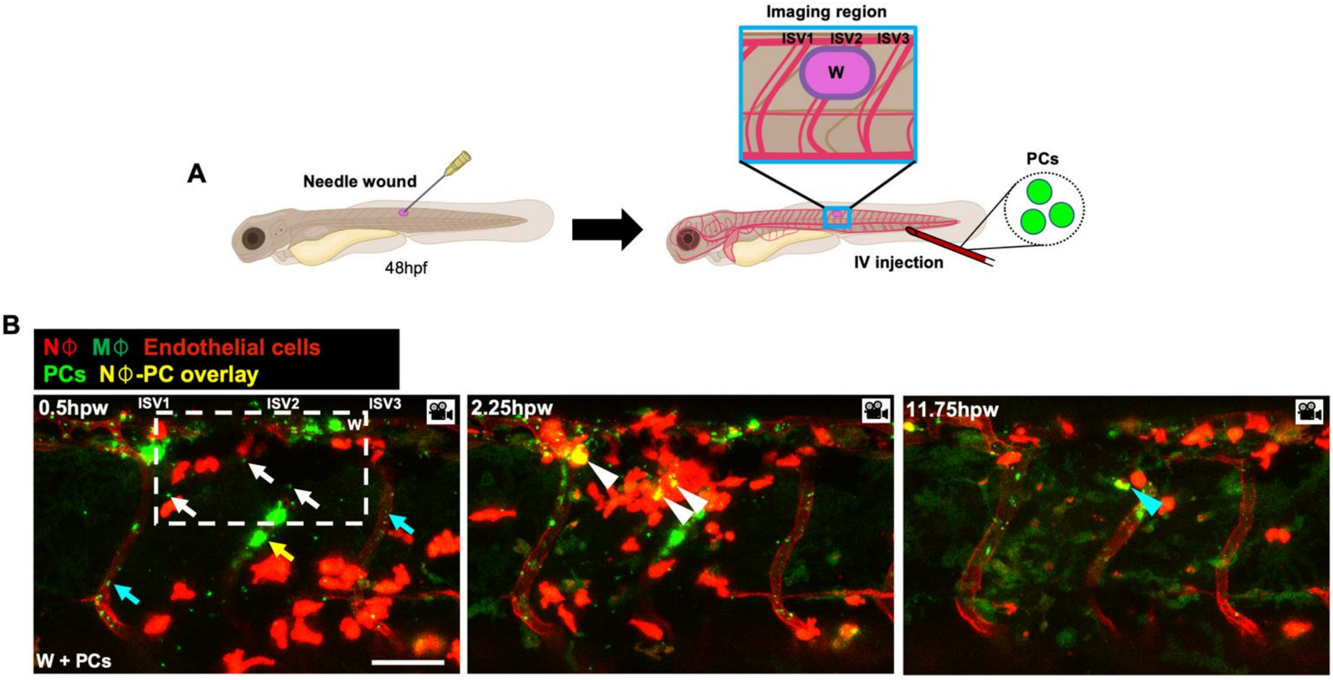

Fig. S1. Protocells can also be taken up by wound neutrophils. (A) Schematic for experiments in (B) showing wound, injection site and imaging regions (blue boxes). **(B)** Confocal images from a time-lapse movie of a wounded Tg(*lyz:DsRed*;*mpeg1:FRET;kdrl:mCherry-CAAX*) larva after injection with FITC-protocells (bright green dots), showing protocells at the wound region (white dashed box) outside vessels (dim red endothelial cells, white arrows), accumulated inside of damaged ISV2 (yellow arrow), flowing within intact ISV1,3 (cyan arrows), inside neutrophils (bright red cells, white arrowheads) or inside macrophages (dim green cells, cyan arrowhead) at 0.5, 2.25 and 11.75 hpw. ISV=intersegmental vessel; MΦ=macrophages; NΦ=neutrophils; W=wound. Scale bars=50 µm.

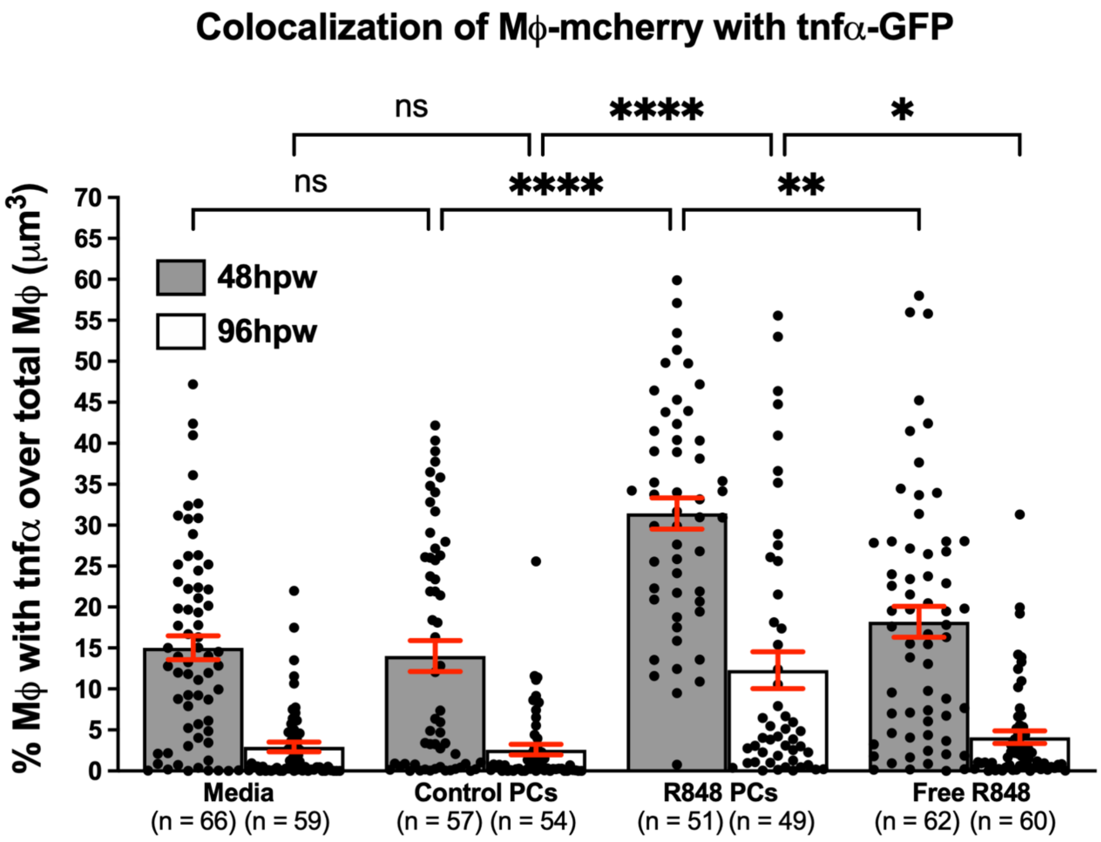

Fig. S2. Injected R848-protocells trigger a higher tnfα expression in larval wound macrophages than free R848. Graph showing percentage of tnfα-positive macrophages after each treatment. Data from "Control PCs" and "R848 PCs" are also represented in Fig. 2G. Same concentration of R848 is used in"R848 PCs" and "Free R848". Each dot in the graph represents one fish. Error bars are mean±s.e.m. ns, not significant; *P<0.05; **P<0.01; ****P<0.0001 (Kruskal–Wallis test with Dunn's multiple comparison post-test). MΦ=macrophages; n=number of fish.

Journal of Cell Science • Supplementary information

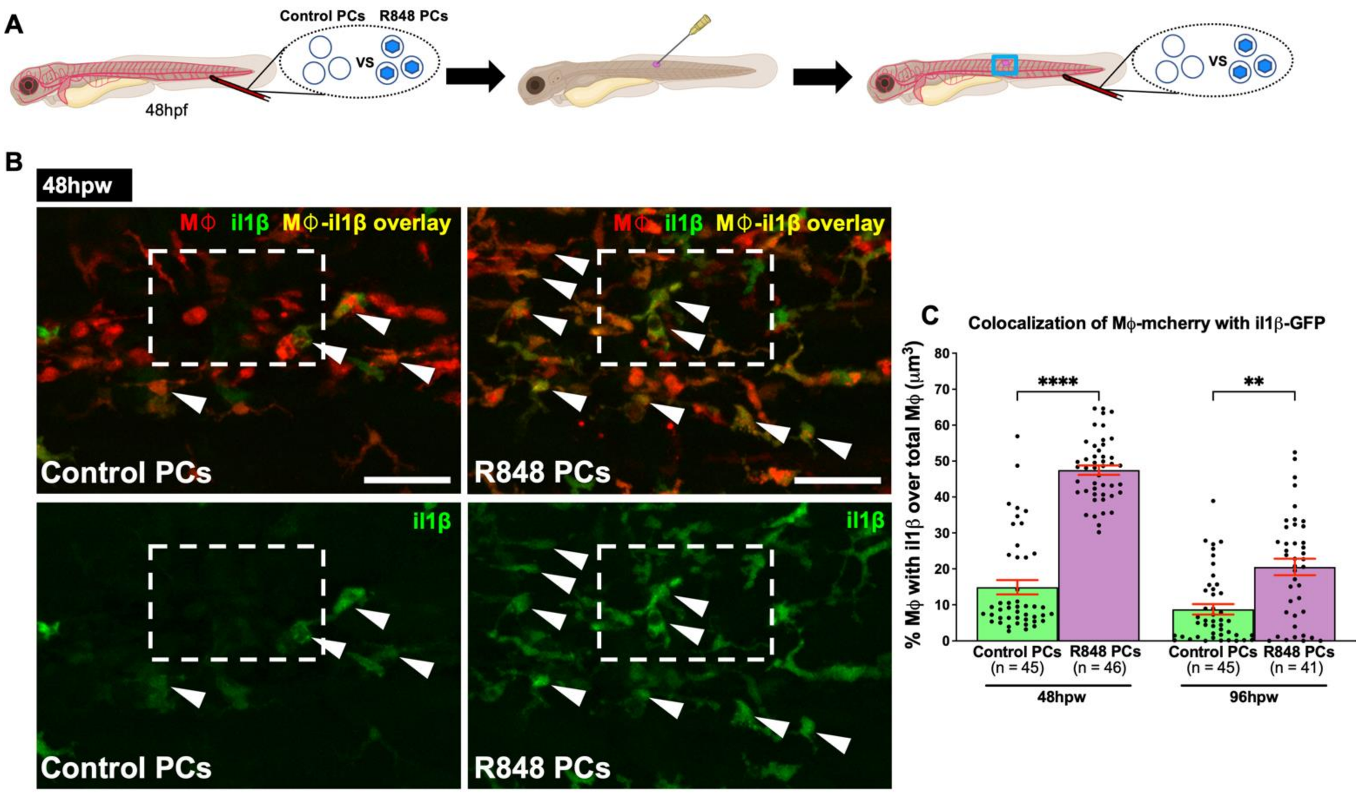

Fig. S3. Uptake of R848-protocells enhances il1β expression in larval wound macrophages. (A) Schematic for experiments in (B,C) showing wounds, injection sites and imaging region (blue box). **(B)** Confocal images of wounded Tg(*mpeg1:mCherry;il1β:GFP*) larvae showing il1β-positive macrophages (white arrowheads) after each protocell treatment. White dashed boxes indicate wound sites. **(C)** Graph showing percentage of il1β-positive macrophages. Each dot in the graph represents one fish. **P<0.01; ****P<0.0001 (unpaired two–sided Mann–Whitney test). n=number of fish. Scale bars=50 μm.

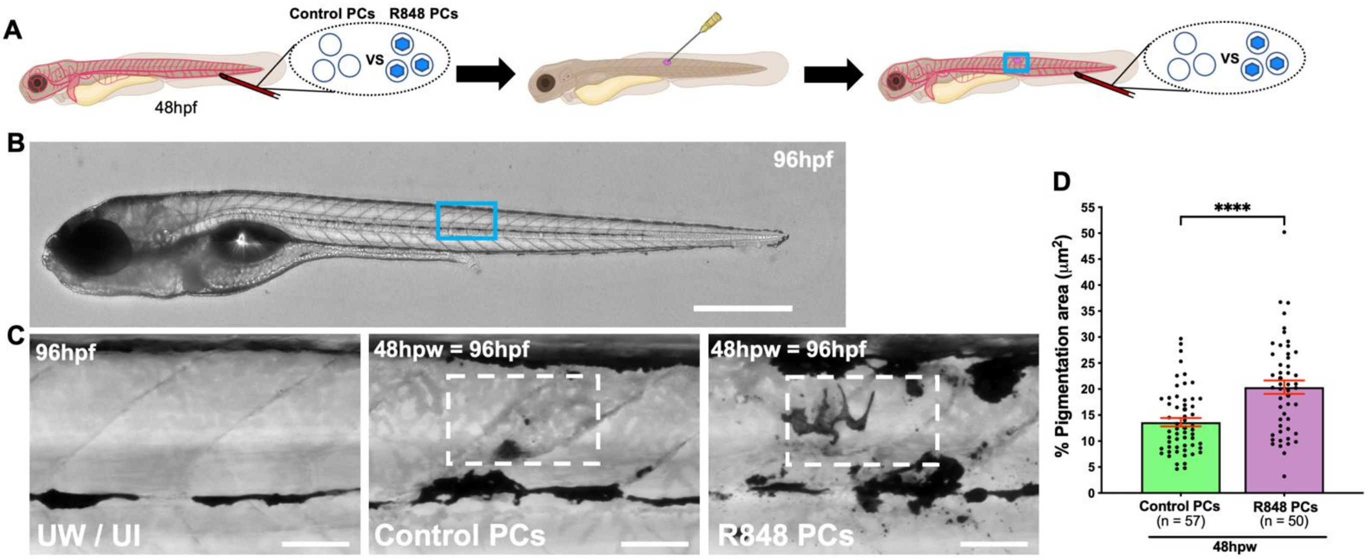

Fig. S4. R848-protocell reprogramming of macrophages leads to hyperpigmented wounds. (A) Schematic for experiments in (B-D) showing wounds, injection sites and imaging region (blue box). **(B)** Confocal image of a whole WT larva (pigmentation in black). Blue box indicates imaging region. **(C)** High magnification views showing unwounded/uninjected (UW/UI) or wounded larvae after each protocell treatment. White dashed boxes indicate wound sites. **(D)** Graph showing percentage of pigmentation area. Each dot in the graph represents one fish. ****P<0.0001 (unpaired two–sided Mann–Whitney test). n=number of fish. Scale bars=500 µm (B), 50 µm (C).

Journal of Cell Science • Supplementary information

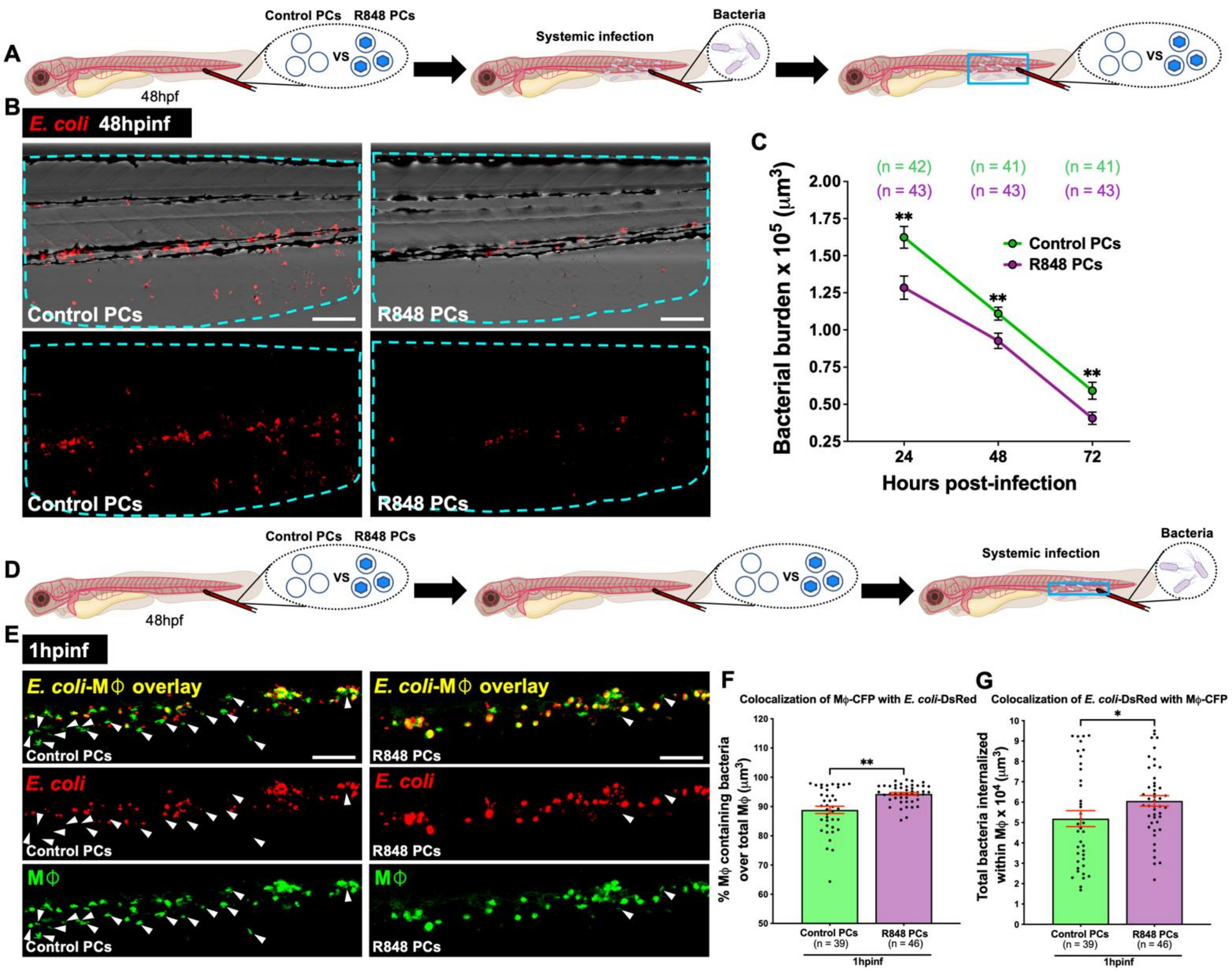

Fig. S5. Enhanced uptake and killing of bacteria by R848-protocells-reprogrammed macrophages. (A,D) Schematics for experiments in (B,C,E-G) showing injection sites and imaging regions (blue boxes). **(B)** Confocal images of infected WT larvae showing bacteria (red) after each protocell treatment. Cyan dashed lines indicate analyzed regions. **(C)** Graph showing bacterial burden. **(E)** Confocal images of infected Tg(*mpeg1:FRET*) larvae showing macrophages with (yellow) or without (green, white arrowheads) internalised bacteria (red) after each protocell treatment. **(F,G)** Graphs showing percentage of macrophages containing bacteria (F) or total bacteria internalized within macrophages (G). Each dot in graph (C) represents the mean of all fish analyzed, and in graphs (F,G) one fish. *P<0.05; **P<0.01 (unpaired two–sided Mann–Whitney test). MΦ=macrophages; n=number of fish. Scale bars=100 µm.

Table S1. Sequence of primers for qPCR assays used in this study. Primers were chosen from PrimerBank (Spandidos et al., 2010) unless otherwise referenced. F=forward; R=reverse.

Gene	Species	Primer sequence (5'-3')		Source	
				PrimerBank ID	Reference
EF1α	Human	F	TGTCGTCATTGGACACGTAGA		(Gurevich et al., 2018)
		R	ACGCTCAGCTTTCAGTTTATCC		
IL1β	Human	F	ATGATGGCTTATTACAGTGGCAA	27894305c1	
		R	GTCGGAGATTCGTAGCTGGA		
IL6	Human	F	AAGCCAGAGCTGTGCAGATGAGTA		(Shinriki et al., 2009)
		R	TGTCCTGCAGCCACTGGTTC		
TNFα	Human	F	CGCTCCCCAAGAAGACAG		(Gurevich et al., 2018)
		R	AGAGGCTGAGGAACAAGCAC		
IL10	Human	F	TCAAGGCGCATGTGAACTCC	24430216c2	
		R	GATGTCAAACTCACTCATGGCT		
MRC1	Human	F	TTCAGAAGGTTTTACTTGGAGTGA		(Gurevich et al., 2018)
		R	TCTCCATAAGCCCAGTTTTCA		

Journal of Cell Science • Supplementary information

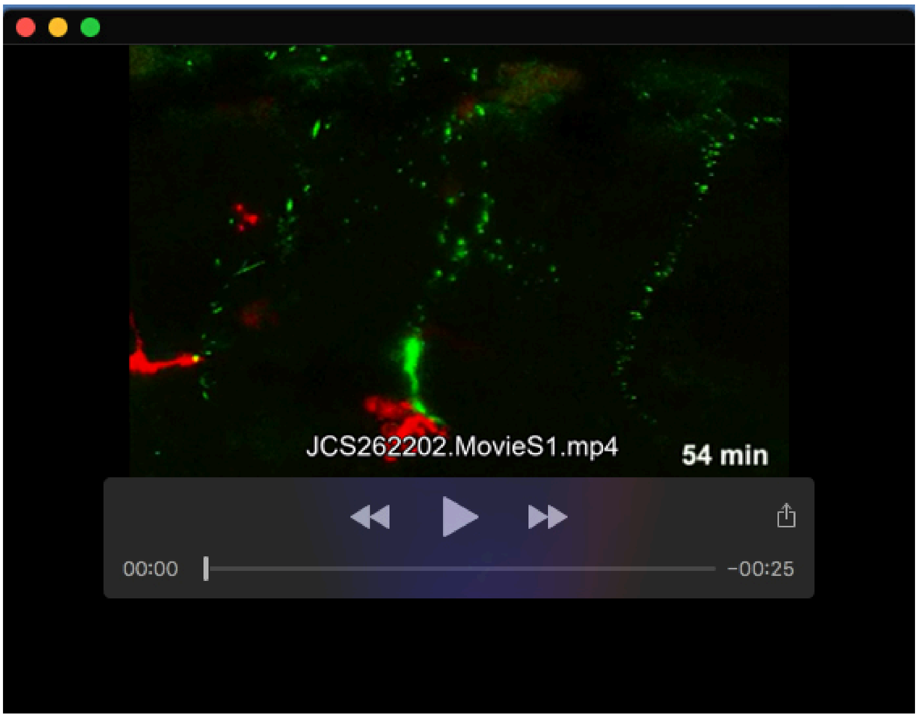

Movie 1. Macrophages effectively taking up protocells at the wound site. Time-lapse confocal movie showing uptake of protocells (green dots) by wound-recruited macrophages (red cells) in a wounded Tg(*mpeg1:mCherry*) larva at 0.5 hours post-wounding after systemic protocell injection. Note that macrophages can take up protocells accumulated at the damaged intersegmental vessel and those that leaked from this vessel towards the wound site.

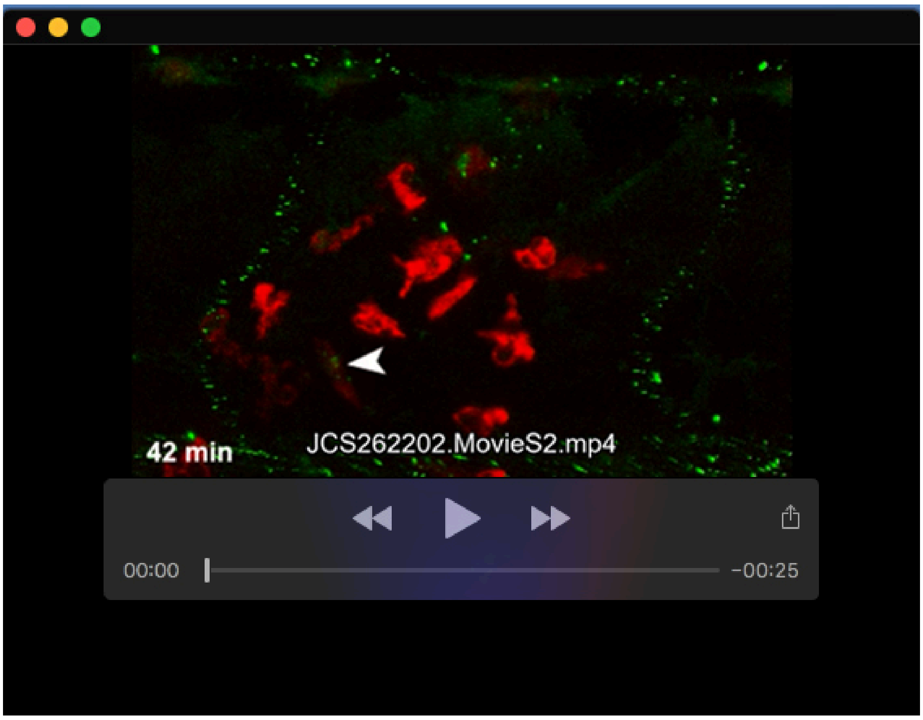

Movie 2. Protocell-loaded macrophages migrating towards the wound site. Time-lapse confocal movie in a different Tg(*mpeg1:mCherry*) larva showing multiple macrophages (red cells) with already internalized protocells (green dots, white arrowheads) prior to their migration towards the wound.

Journal of Cell Science • Supplementary information

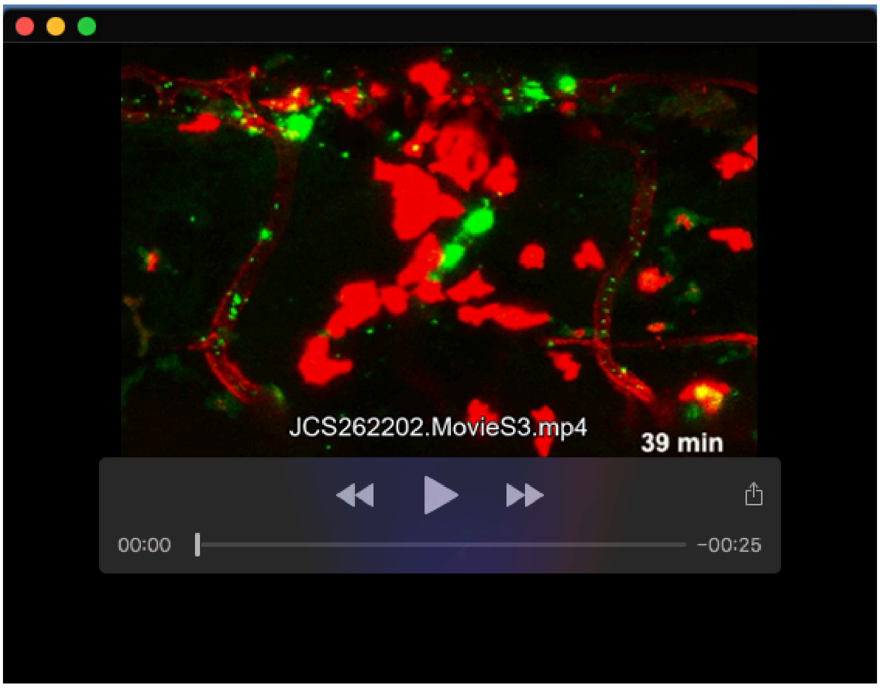

Movie 3. Neutrophils effectively taking up protocells at the wound site. Time-lapse confocal movie showing uptake of protocells (bright green dots) by wound-recruited neutrophils (bright red cells, white arrowheads) in a wounded Tg(*lyz:DsRed*;*mpeg1:FRET;kdrl:mCherry-CAAX*) larva at 0.5 hours post-wounding after systemic protocell injection. Note that this movie also shows how wound-recruited macrophages (dim green cells) can take up protocells at the wound site (cyan arrowhead) and how neutrophils, similarly to macrophages, already contain protocells prior to their recruitment to the wound (yellow arrowhead).

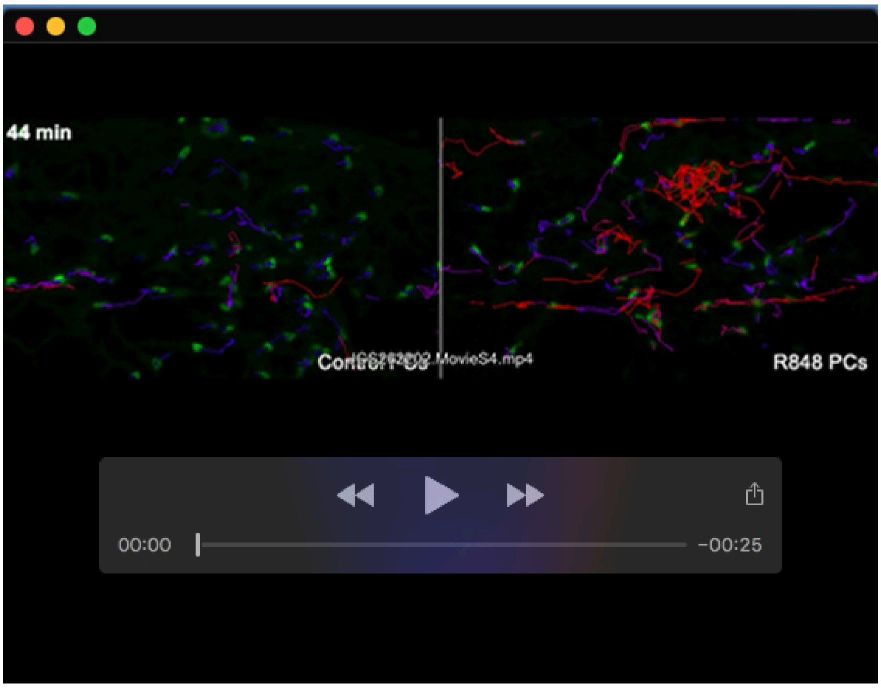

Movie 4. R848-protocell treatment increases macrophage velocity at the wound site. Time-lapse confocal movies showing tracks of macrophage nuclei (green) at the wound site in wounded Tg(*mpeg1:FRET*) larvae at 48 hours post-wounding after each protocell treatment. Note that warm and cold track colours indicate faster and slower macrophage velocity, respectively.

Journal of Cell Science • Supplementary information

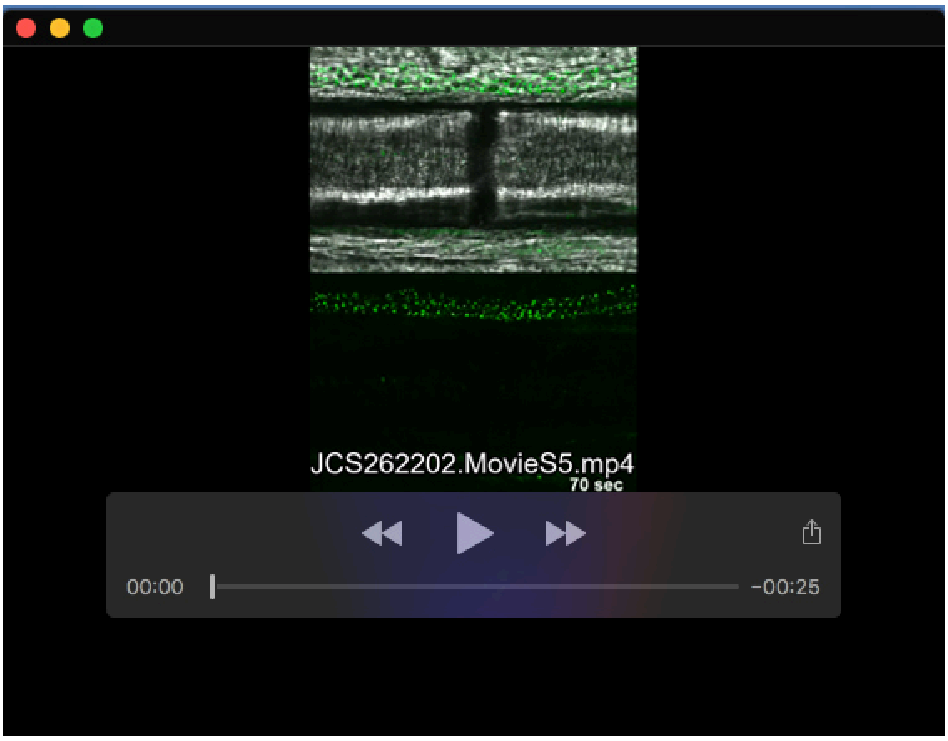

Movie 5. Retro-orbital injection effectively delivers protocells to adult caudal fin vessels. Time-lapse confocal movie showing free-circulating protocells (green dots) flowing through caudal fin vasculature in a WT adult fish at 1 hour after injection via retro-orbital administration.

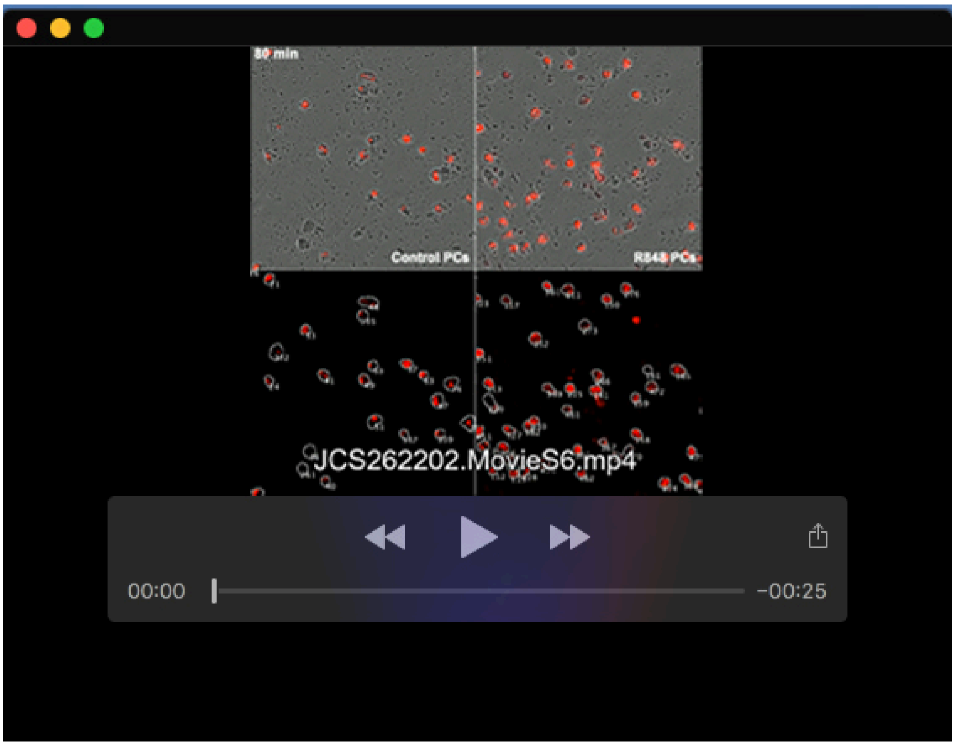

Movie 6. R848-protocell treatment increases bacterial uptake by macrophages. Time-lapse Incucyte movies of zoomed-in regions from wells containing cultured human macrophages incubated with bacterial bioparticles (red) after each protocell treatment. Note that white outlines indicate detected macrophages and white numbers indicate the order of appearance for detected macrophages.

Supplementary References

Gurevich, D. B., Severn, C. E., Twomey, C., Greenhough, A., Cash, J., Toye, A. M., Mellor, H. and Martin, P. (2018). Live imaging of wound angiogenesis reveals macrophage orchestrated vessel sprouting and regression. *EMBO J.* **37**, e97786.doi:10.15252/embj.201797786.

Shinriki, S., Jono, H., Ota, K., Ueda, M., Kudo, M., Ota, T., Oike, Y., Endo, M., Ibusuki, M., Hiraki, A., et al. (2009). Humanized Anti-Interleukin-6 Receptor Antibody Suppresses Tumor Angiogenesis and In vivo Growth of Human Oral Squamous Cell Carcinoma. *Clin. Cancer Res.* **15**, 5426–5434.doi:10.1158/1078-0432.CCR-09-0287.

Spandidos, A., Wang, X., Wang, H. and Seed, B. (2010). PrimerBank: a resource of human and mouse PCR primer pairs for gene expression detection and quantification. *Nucleic Acids Res.* **38**, D792–D799.doi:10.1093/NAR/GKP1005.

Journal of Cell Science • Supplementary information
