# Supplementary material for: Reprogramming macrophages with R848-loaded artificial protocells to modulate skin and skeletal wound healing
Source: J Cell Sci. 2024 Aug 29;137(16):jcs262202. doi: 10.1242/jcs.262202 (PMC11385641; doi:10.1242/jcs.262202)
Supplement: Supplementary file 1 [file joces-137-262202_review_history.pdf]

Reprogramming macrophages with R848-loaded artificial protocells to modulate skin and skeletal wound healing

Paco Lopez-Cuevas, Tiah CL Oates, Qiao Tong, Lucy M. McGowan, Stephen J. Cross, Can Xu, Yu Zhao, Zhuping Yin, Ashley M. Toye, Asme Boussahel, Chrissy L Hammond, Stephen Mann and Paul Martin
DOI: 10.1242/jcs.262202

Editor: Caroline Hill

Review timeline

Original submission:	16 April 2024
Editorial decision:	10 June 2024
First revision received:	4 July 2024
Accepted:	15 July 2024

Original submission

First decision letter

MS ID#: JOCES/2024/262202

MS TITLE: Reprogramming macrophages with R848-loaded artificial protocells to modulate zebrafish skin and skeletal wound healing

AUTHORS: Paco Lopez-Cuevas, Tiah CL Oates, Qiao Tong, Lucy M McGowan, Stephen J Cross, Can Xu, Yu Zhao, Zhuping Yin, Ashley M Toye, Asme Boussahel, Chrissy L Hammond, Stephen Mann, and Paul Martin

We have now reached a decision on the above manuscript.

To see the reviewers' reports and a copy of this decision letter, please go to: https://submit-jcs.biologists.org and click on the 'Manuscripts with Decisions' queue in the Author Area. (Corresponding author only has access to reviews.)

As you will see, the reviewers raise a number of substantial criticisms that prevent me from accepting the paper at this stage. They suggest, however, that a revised version might prove acceptable, if you can address their concerns. If you think that you can deal satisfactorily with the comments and criticisms on revision, I would be very pleased to see a revised manuscript.

Please ensure that you clearly highlight all changes made in the revised manuscript. Please avoid using 'Tracked changes' in Word files as these are lost in PDF conversion.

I should be grateful if you would also provide a point-by-point response detailing how you have dealt with the points raised by the reviewers in the 'Response to Reviewers' box. Please attend to all of the reviewers' comments. If you do not agree with any of their criticisms or suggestions please explain clearly why this is so.

Reviewer 1

Advance summary and potential significance to field

© 2024. Published by The Company of Biologists under the terms of the Creative Commons Attribution License (https://creativecommons.org/licenses/by/4.0/).

The manuscript by Lopez-Cuevas et al. investigated the effect of macrophage reprogramming by protocells loaded with R848. Protocells are taken up by macrophages and neutrophils and delayed healing, but they are beneficial when the wound is infected. Furthermore, the protocell can deliver R848 to human macrophages and induce their polarization to M1, too. Overall, this work is interesting and provides proof of concepts of using protocell-R848 to target macrophage and alter wound healing. However, there are several limitations that should be addressed.

Comments for the author

Major concerns:
1. The manuscript is written in a short report format. However, given its current contents, it is more suitable as a regulator research paper.
The introduction needs to be expanded. It lacks sufficient background on the protocell. How is the protocell formulated? How specific is protocell-mediated delivery? Is the uptake restricted to immune cells? Does the protocell itself cause inflammation in the absence of wound or tissue damage? What is the maximal safety dose of protocell for zebrafish?
The figures are cramped, and the fonts in bar graphs are too small to read. It is recommended that figures 1 and 2 be split into 2 figures.
The results session also lacks sufficient introduction and rationale for some of the experiments, for example, the relevance of cell extrusion in wounding and the rationale and method for visualization callus and osteoclasts in bone healing.
There is also a lack of sufficient discussion on how macrophages alter the overall inflammation response, especially during wound response, the biological significance of the altered vascular network, the discrepancy of IL-10 transcript and protein levels, and the benefit of using protocells to deliver R848 over systemic delivery or other targeted delivery methods.
2. Overall, the manuscript lacks disclosure of the biological repeats for all the experiments and the total fish number quantified.
3. The method of determining the concentration of R848 in protocell is not included in the manuscript. Some experiment procedures lack sufficient details for replication. For example, the concentration of MS-222 used is not provided.
4. Figure 1G lacks error bars. The results indicated low-level leakage, rather than no leak at all, as indicated in the results section.
5. The quality of the Krt4:GPF in Figure 2K and R is poor, preventing the observation of individual cells. It is not clear what conclusion can be drawn from 2Ki.
6. There is no justification for how the dose of protocell was determined for use in human macrophages. Will protocells cause macrophage death? Will macrophage activation, for example by LPS, alter the protocell uptake? Bacteria killing rather than phagocytosis is a more relevant functional readout.
7. The proinflammatory phenotype in macrophages in zebrafish is defined as TNFalpha positive. It would be beneficial to include an independent method to reflect the proinflammatory status such as cytokine qRT in zebrafish.
Minor concerns:
1. The title should be modified to reflect the context of the paper, including human macrophages.
2. The manuscript should be checked carefully for typos. For example, the protocell is misspelled several times.
3. Abbreviations, such as BSA, are not defined.
4. Figure 2 legend is not in a sequential order and thus is hard to follow.

<u>Reviewer 2</u>

Advance summary and potential significance to field

The manucript « Reprogramming macrophages with R848-loaded artificial protocells to modulate zebrafish skin and skeletal wound healing” by Paco Lopez-Cuevas, Tiah CL Oates, Qiao Tong, Lucy M McGowan, Stephen J Cross, Can Xu, Yu Zhao, Zhuping Yin, Ashley M Toye, Asme Boussahel, Chrissy L Hammond, Stephen Mann and Paul Martin reports the reprogramming of zebrafish macrophages toward a proinflammatory and bactericidal phenotype using R848 loaded protocells. Their results

© 2024. Published by The Company of Biologists under the terms of the Creative Commons Attribution License (https://creativecommons.org/licenses/by/4.0/).

suggest that human macrophages do behave the same. They also show that this proinflammatory reprogramming of macrophages partially impairs wound healing.

The reported work is interesting, well done, correctly discussed, and opens new perspectives for immunologists working with whole animals. It may have implications in the clinic.

Comments for the author

The reported work is interesting, well done and correctly discussed, but the figures should definitively be improved to help the reader understand what exactly has been done, what exactly has been observed and how it may substantiate the conclusions of the authors.
I include many minor criticisms some related to the main text, most related to the figures, that should be addressed by the authors before the article would be accepted.

In order to avoid possible confusion, it is absolutely necessary that the transgenic lines be listed in the “Zebrafish lines and maintenance” with their exact names, as for example TgBAC(il-1ß:eGFP)sh445 or TgBAC(tnfa:GFP)pd1028. This is critical in the case of what the authors call “Tg(mpeg1:FRET)” that is a jargon name for the Tg(mpeg1.1:CFP-DEVD-YFP)sh266 transgenic line. Please do not use the jargon “M-FRET” in figure S4.

Line 164: “…. cells and small and circular krt9”, did the authors really mean krt9? …. rather than krt19?

Figure1: Please explain the abbreviation “IVS” and “TNFa+ve”. Please check for “ve” in all figures and find something more self-explanatory.

Fig1I: The whole panel 1I is labelled “Macrophages tnfa Overlay” and this is misleading. Each individual panel of Fig1I should be labeled either “overlay”(upper left and upper right) or “tnfa”(lower left and lower right) while “48hpw” should apply to the Wole panel 1I.

Fig S2b: Please explain “IL1b+ve”. Similar to what I wrote for fig 1I, “48hpw” refers to the four images, and should be written where “Macrophages ILb Il1b+ve Macrophages” is written while “IL1b” should be written on both lower panels and the whole on the upper panels.

Line 164: “cells and small and circular krt9”, did the authors really mean krt9? …. rather than krt19?

Figure 2: Since the time frame of the experiment (wound at 48hpf) is not highlighted in fig2, I suggest that “96hpw” be replaced by “96hpw = 144 hpf”, “120 hpw” be replaced by “120 hpw= 168 hpf”, “48hpw” be replaced by “48hpw = 96hpf” and “72hpw” be replaced by “72hpw = 120 hpf”.
Please use larger fonts for the legend of the axis of all diagrams and figures, C, D E, F, I, L, M, N, T, U and V.
Please indicate time point in each image.

Figure 2, K: If I understand well, Ki and Kii are individual color channels of K “848 PCs”. Since this is really not self-explanatory, I think the authors should find a way for this to be obvious for the reader. It could be two arrows coming from the dotted rectangle in “848 PCs” to Ki and Kii.
Figure 2, Ki: why is the green present only within the dotted rectangle while the red is present all over Kii and green present in the whole panel in the upper panels?

Figure 2P: Time points are barely readable! It is really not clear what 2Pi, 2Pii and 2Piii are.

Lines 146-154 and FigS3: The experimental design is not well presented. We have no indication of the timing of the wound (48hpf ?) nor of the timing if PCs injection. This should be clearly stated both in the text and in the figure (see below). Schematic for the experimentation should be included in FigS3.

FigS3: The developmental stage of the embryo should be indicated in all images (A plus B).
On the central and right images of S3B, I suggest that the authors write “96hpf=48hpw). Please include a schematic for the experimentations.

© 2024. Published by The Company of Biologists under the terms of the Creative Commons Attribution License (https://creativecommons.org/licenses/by/4.0/).

Figure 3E: The whole panel E is labelled “Macrophages PCs Overlay” and this is misleading. Each individual panel of Fig3E should be labeled either”transmission”(upper left) “macrophages”(lower right) or ‘PCs”(upper right) or “overlay”(lower left) while “11hpfr/3hpi” should apply to the wole panel 3E.

Figure 3G & 3H: Same as for 3E, please correctly label each individual panel.

Figure 3J-N : Text and numbers are unreadable, please use larger fonts for all text and all numbers even for the axis numbering.

Line 202: we live-stained Tg(osx:mCherry) adult fish (with RFP-tagged osteoblasts). Should be “we live-stained Tg(osx:mCherry) adult fish (with mCherry-tagged osteoblasts).”

Lines 206-209: The authors should refer to the TRAP labelling of osteoclasts.

FigS4D: Please write “systemic infection” similar to what is written in S4A and S4H

Fig S4I: Similar critic as for Fig1I and Fig3E.

FigS4I: “48hpinf” applies to the whole panel or just to the upper left image?

Fig 4: All texts are un readable, please use a larger font size.

Fig4B: the IL1b panel highlight the fact that a linear scale is not appropriate to display value ranging more than on log. It is necessary in such a case to use a log scale. Please modify!

Fig 4C: Same as for 4B, it is necessary to use a log scale for all panels… except (perhaps) for IL1RA.

Fig 4D: “40mpinf”” refers to all 4 images, please write “40mpinf” nest to E.coli.

Line 274: “resuspended in 2% PVP40 solution (Sigma) and phenol red (Sigma).” Please explain what exactly you mean by “2% PVP40 solution (Sigma)”? … is it buffered? … and why you use PVP.

Line 327: “Larvae were anaesthetized at 48 hpf in MS-222 (Sigma)”. MS-222 is a commercial reference, not a product, I suggest that the authors write ““Larvae were anaesthetized at 48 hpf using tricaine {please indicate the final concentration} (Sigma MS-222)”

Line 331, 340, 346, 366, 371: Same as for line 327.

Line 347: “blunt-ended glass capillary tube”. Reference and/or details?

Line 353: “pH adjusted to 8”… using what?

Lines 481-483: It is not normal that datasets availability would depend on a person. They should be available using DOIs on public/permanent repositories.

<u>Reviewer 3</u>

Advance summary and potential significance to field

In this work, Lopez-Cuevas et al. present compelling data on the use of artificial protocells and a TLR7/8 agonist to modulate wound healing in two zebrafish wounding models with some extension to human macrophages. This work follows up a 2022 paper from the same group in which an anti-miR223 cargo is delivered in the context of an in vivo tumor model. There are nicely characterized effects on macrophage profile and wound healing processes, expertly characterized throughout the paper, and, finally, the authors demonstrate an effect on bacterial phagocytosis of human peripheral blood-derived macrophages. The breadth of assays used (and careful characterization therein) suggests that this strategy may have general utility, and the retroorbital injections in

© 2024. Published by The Company of Biologists under the terms of the Creative Commons Attribution License (https://creativecommons.org/licenses/by/4.0/).

adults followed by bone fracture were particularly impressive. Overall there are two major additions that would considerably strengthen the manuscript.

Comments for the author

1) The protocells are a very nice and novel approach, but I couldn't find any direct comparison to just administering the same drug in similar quantities, either through injection or even immersion. This would be very helpful to formally demonstrate potential advantages of the protocells.

2) The assumption is that the drug acts as a TLR7/8 agonist (and that these molecules might normally be inaccessible to the drug). This should really be tested for specificity in some way other than pro-inflammatory output. I recognize that there are multiple TLR7/8 paralogues in zebrafish so a comprehensive genetic approach would be difficult, but there should be some way to build evidence for or against, particularly as I assume that this target has not been demonstrated before in zebrafish. Multiplexed CRISPR with assessment of crispants? Perhaps an antagonist? Or perhaps interfering with TLR7/8 via something like the unc93 pathway? Since most of the paper focuses on zebrafish, I think it is important to at least attempt to show some specificity there, but lower priority conceptually could go to the human system and inhibition there (siRNAs?).

The introduction is short, as is the discussion. All fine, particularly for a short report format, but I do think there is an opportunity to orient the reader a bit more about protocells (advantages, delivery, etc) as well as more robust discussion of how strongly the TLR7/8 activation should be pursued as a therapeutic strategy with pitfalls or whether this manuscript represents further proof of principle in the context of infected wounds. For the therapeutic discussion generally, may be worth commenting on the pre-administration that is used in the manuscript to see full effect. And since many of the assays show impairments with protocells, it might be nice to have at least some of Figure S4 (enhanced uptake and killing of bacteria upon PC treatment) as part of a main figure or a standalone figure.

Finally, just a general compliment about the quality of the imaging and the movies!

First revision

Author response to reviewers' comments

Please see 'Rebuttal Letter' document in Supplemental Files. Any changes to the text of the MS are highlighted in green.

Dear Caroline Hill - please find our resubmitted MS - "Reprogramming macrophages with R848-loaded artificial protocells to modulate skin and skeletal wound healing ". We were very pleased that all three of your referees seemed to like it, but at least two of them suggested expanding various sections to include extra details and splitting figures etc. I hope you don't consider this presumptuous, but we too felt that we had somewhat constrained ourselves by going for the Short Report format, and so our resubmission is now expanded to an Article type paper instead.

Below we address, in a point-by-point fashion, each of the referees' concerns and how we have dealt with them in our revised MS. Any changes to the text of the MS are highlighted in green.

Reviewer 1 Advance Summary and Potential Significance to Field:
The manuscript by Lopez-Cuevas et al. investigated the effect of macrophage reprogramming by protocells loaded with R848. Protocells are taken up by macrophages and neutrophils and delayed healing, but they are beneficial when the wound is infected. Furthermore, the protocell can deliver R848 to human macrophages and induce their polarization to M1, too.
Overall, this work is interesting and provides proof of concepts of using protocell-R848 to target macrophage and alter wound healing. However, there are several limitations that should be addressed.

© 2024. Published by The Company of Biologists under the terms of the Creative Commons Attribution License (https://creativecommons.org/licenses/by/4.0/).

Reviewer 1 Comments for the Author:

Major concerns:

1. The manuscript is written in a short report format. However, given its current contents, it is more suitable as a regulator research paper. The introduction needs to be expanded. It lacks sufficient background on the protocell. How is the protocell formulated? How specific is protocell-mediated delivery? Is the uptake restricted to immune cells?
We agree with this and since another reviewer also has encouraged expanding the paper to add more detail, we have now expanded the Intro, Results and Discussion sections of the MS to fit it into a research paper format. Regarding expansion of protocell background, we have addressed this in the new Results section: lines 111-113 for the protocell formulation; and in the new Intro section: lines 77-78 for protocell delivery/uptake specificity.

Does the protocell itself cause inflammation in the absence of wound or tissue damage?
We did not observe any changes in the number of inflammatory macrophages in fish injected with protocells versus control media in unwounded fish. This was reported in Fig. 5R of our previous study López-Cuevas et al. (2022), *Advanced Science*. We have also added an additional experiment to our revised MS, with our wounded fish model in Fig. S2 that shows no differences in the proportion of tnfα-positive macrophages between “protocells” and control “media” groups (lines 169-171). Together these data suggest that protocells, in themselves, do not trigger a significant inflammatory response.

What is the maximal safety dose of protocell for zebrafish?
We have previously performed titration experiments (López-Cuevas et al., 2022) (Fig. 2) and showed that the maximal safety dose of protocells for zebrafish injection (via intravenous delivery) is 2 nL at a concentration of 1.25×10^7 protocells/μL. Any injection with a higher concentration resulted in clogging of the zebrafish blood circulation (data not shown). We have added this new information in M&M (lines 478-479).

The figures are cramped, and the fonts in bar graphs are too small to read. It is recommended that figures 1 and 2 be split into 2 figures.
Sorry about this, we agree; both the text and figures were cramped by our trying to adhere to the Short Report format. Font size has now been increased in all graphs to make it more readable. We have split both of the previous Figs. 1 and 2, which now become the new Figs. 1-4.

The results session also lacks sufficient introduction and rationale for some of the experiments, for example, the relevance of cell extrusion in wounding and the rationale and method for visualization callus and osteoclasts in bone healing.
As explained above we have now expanded our Introduction and so hope that these issues are clearer now. The relevance of cell extrusion in wounding and its significance in the context of our results is now described in lines 232-234 of the Results section. Regarding callus and osteoclasts during bone healing, we have explained the rationale and methods for their visualization in adult zebrafish from line 271 to 285.

There is also a lack of sufficient discussion on how macrophages alter the overall inflammation response, especially during wound response, the biological significance of the altered vascular network, the discrepancy of IL-10 transcript and protein levels, and the benefit of using protocells to deliver R848 over systemic delivery or other targeted delivery methods.
We have added discussion for each of these points:
-Macrophage alteration of overall inflammation response during wound response: lines 147- 153
-Biological significance of the altered vascular network: lines 187-189
-Discrepancy of IL-10 transcript and protein levels: lines 308-310
-Benefit of using protocells to deliver R848 over other delivery methods: lines 100-107 and lines 338-341.

2. Overall, the manuscript lacks disclosure of the biological repeats for all the experiments and the total fish number quantified.
We have addressed this at the end of the “Statistical analysis” section (lines 630-631). Total fish

© 2024. Published by The Company of Biologists under the terms of the Creative Commons Attribution License (https://creativecommons.org/licenses/by/4.0/).

number (n) used for quantifications is included in the graphs for each experiment in all Figs. This is also mentioned in line 628 and in the legends for each Fig.

3. The method of determining the concentration of R848 in protocell is not included in the manuscript. Some experiment procedures lack sufficient details for replication. For example, the concentration of MS-222 used is not provided.
Tricaine (MS-222) concentration used in our fish experiments and the method used for determining R848 concentration in protocells are now included in the M&M, in lines 476, 481, 490, 496, 516 and 521, and in lines 406-408, respectively.

4. Figure 1G lacks error bars. The results indicated low-level leakage, rather than no leak at all, as indicated in the results section.
Sorry. Missing error bars in the old Fig. 1G (now new Fig. 2C), have now been incorporated, and the description of these data corrected in line 143.

5. The quality of the Krt4:GPF in Figure 2K and R is poor, preventing the observation of individual cells. It is not clear what conclusion can be drawn from 2Ki.
We have enlarged these images and split them into single channels to aid visualisation at the cell level. Panel in 2Ki is a MAX projection image (from the wound region of “R848 PCs” fish indicated by blue arrows) and it contains the outlines for the high-fluorescent krt4:GFP regions that have been automatically drawn, based on a fluorescence intensity threshold, by our MIA software. This image analysis method allowed us to quantify the volume enclosed by “extruded” krt4 cells post-wounding.

6. There is no justification for how the dose of protocell was determined for use in human macrophages.
We previously tested different protocell concentrations in human macrophage cultures (López-Cuevas et al., 2022) (Fig. 6B,D) and selected the protocell dose that was rendering the highest macrophage uptake without apparent toxicity. This has now been clarified in the M&M (line 437).

Will protocells cause macrophage death?
We routinely quantified macrophage numbers before and after protocell administration for all our human macrophage experiments, and even at the highest protocell concentration used (100 protocells per macrophage), we did not observe macrophage deaths above the background levels.

Will macrophage activation, for example by LPS, alter the protocell uptake?
We have not addressed this question directly with LPS, but we believe that macrophage activation will probably improve protocell uptake because our own results (presented in this paper) show that activated macrophages (triggered by R848-protocell treatment) exhibit enhanced uptake of bacterial particles.

Bacteria killing rather than phagocytosis is a more relevant functional readout.
We used bacterial particles instead of live bacteria to avoid possible cross-contamination and therefore we felt that phagocytosis assays rather than bacterial killing assays were more appropriate in this instance.

7. The proinflammatory phenotype in macrophages in zebrafish is defined as TNFalpha positive. It would be beneficial to include an independent method to reflect the proinflammatory status such as cytokine qRT in zebrafish.
We used both tnfalpha and il1beta zebrafish reporters as readouts for pro-inflammatory macrophages because it is a well-established and rapid method to determine phenotype of inflammatory macrophages, and given that fish can be kept alive after each imaging time point, this makes it easier to follow dynamics in gene expression compared to fixed time points in qRT assays.

Minor concerns:

1. The title should be modified to reflect the context of the paper, including human macrophages.
Zebrafish are the primary, pre-clinical model in this study and although our human macrophage work is exciting, the proposed protocell therapy is still far from in vivo clinical applications. Therefore,

© 2024. Published by The Company of Biologists under the terms of the Creative Commons Attribution License (https://creativecommons.org/licenses/by/4.0/).

we feel that adding the human context in the title would be misleading and may induce the reader to think that we have CLINICAL evidence for the protocell strategy being used in humans. However, we would consider losing the word "zebrafish" from our title if you and the Editor felt this was more appropriate and so as to make it appear not ONLY a zebrafish paper.

2. The manuscript should be checked carefully for typos. For example, the protocell is misspelled several times.

Thanks for spotting this. We have now revised the MS for typos and corrected all misspelled words.

3. Abbreviations, such as BSA, are not defined.

All abbreviations, including BSA, are now defined in their first appearances.

4. Figure 2 legend is not in a sequential order and thus is hard to follow.

We have attempted to do this by changing panel labels.

*****

Reviewer 2 Advance Summary and Potential Significance to Field:
The manucript « Reprogramming macrophages with R848-loaded artificial protocells to modulate zebrafish skin and skeletal wound healing" by Paco López-Cuevas, Tiah CL Oates, Qiao Tong, Lucy M McGowan, Stephen J Cross, Can Xu, Yu Zhao, Zhuping Yin, Ashley M Toye, Asme Boussahel, Chrissy L Hammond, Stephen Mann and Paul Martin reports the reprogramming of zebrafish macrophages toward a proinflammatory and bactericidal phenotype using R848 loaded protocells. Their results suggest that human macrophages do behave the same. They also show that this proinflammatory reprogramming of macrophages partially impairs wound healing.
The reported work is interesting, well done, correctly discussed, and opens new perspectives for immunologists working with whole animals. It may have implications in the clinic.

Reviewer 2 Comments for the Author:
The reported work is interesting, well done and correctly discussed, but the figures should definitively be improved to help the reader understand what exactly has been done, what exactly has been observed and how it may substantiate the conclusions of the authors.
I include many minor criticisms some related to the main text, most related to the figures, that should be addressed by the authors before the article would be accepted.
In order to avoid possible confusion, it is absolutely necessary that the transgenic lines be listed in the "Zebrafish lines and maintenance" with their exact names, as for example TgBAC(il-1ß:eGFP)sh445 or TgBAC(tnfa:GFP)pd1028. This is critical in the case of what the authors call "Tg(mpeg1:FRET)" that is a jargon name for the Tg(mpeg1.1:CFP-DEVD-YFP)sh266 transgenic line. Please do not use the jargon "M-FRET" in figure S4.
We have added the exact names for all zebrafish lines used. We have corrected M-FRET by M-CFP, to better reflect the Tg(mpeg1.1:CFP-DEVD-YFP)sh266 full name, in Suppl. Fig.

Line 164: "…. cells and small and circular krt9", did the authors really mean krt9? …. rather than krt19?
Sorry for the typo; yes, we did mean "krt19". Thanks; this is corrected now.

Figure1: Please explain the abbreviation "IVS" and "TNFa+ve". Please check for "ve" in all figures and find something more self-explanatory.
We have removed TNFa+ve in all figures and replaced it by M-tnfa. We have added the meaning of ISV (intersegmental vessel) in Fig. 1B panel and legend.

Fig1I: The whole panel 1I is labelled "Macrophages tnfa Overlay" and this is misleading. Each individual panel of Fig1I should be labeled either "overlay"(upper left and upper right) or "tnfa"(lower left and lower right) while "48hpw" should apply to the Wole panel 1I.

Done.

Fig S2b: Please explain "IL1b+ve". Similar to what I wrote for fig 1I, "48hpw" refers to the four images, and should be written where "Macrophages ILb Il1b+ve Macrophages" is written while "IL1b" should be written on both lower panels and the whole on the upper panels.
We have removed IL1b+ve and replaced it by M-IL1b, and applied the suggested changes to the

© 2024. Published by The Company of Biologists under the terms of the Creative Commons Attribution License (https://creativecommons.org/licenses/by/4.0/).

Figure.

Line 164: “cells and small and circular krt9”, did the authors really mean krt9? …. rather than krt19?
Answered above.

Figure 2: Since the time frame of the experiment (wound at 48hpf) is not highlighted in fig2, I suggest that “96hpw” be replaced by “96hpw = 144 hpf”, “120 hpw” be replaced by “120 hpw= 168 hpf”, “48hpw” be replaced by “48hpw = 96hpf” and “72hpw” be replaced by “72hpw = 120 hpf”. Please use larger fonts for the legend of the axis of all diagrams and figures, C, D E, F, I, L, M, N, T, U and V. Please indicate time point in each image.

Done.

Figure 2, K: If I understand well, Ki and Kii are individual color channels of K “848 PCs”. Since this is really not self- explanatory, I think the authors should find a way for this to be obvious for the reader. It could be two arrows coming from the dotted rectangle in “848 PCs” to Ki and Kii.

We have followed your advice and added the arrows.

Figure 2, Ki: why is the green present only within the dotted rectangle while the red is present all over Kii and green present in the whole panel in the upper panels?
We have improved this by showing only the regions within the dotted rectangle for both Ki and Kii.

Figure 2P: Time points are barely readable! It is really not clear what 2Pi, 2Pii and 2Piii are.
We have added a black box as background to the figure to make white timepoint labels readable, and blue arrows to indicate where i, ii and iii panels are from.

Lines 146-154 and FigS3: The experimental design is not well presented. We have no indication of the timing of the wound (48hpf ?) nor of the timing if PCs injection. This should be clearly stated both in the text and in the figure (see below).
Schematic for the experimentation should be included in FigS3.

We have now done all of this, as suggested.

FigS3: The developmental stage of the embryo should be indicated in all images (A plus B).
On the central and right images of S3B, I suggest that the authors write “96hpf=48hpw). Please include a schematic for the experimentations.

Done.

Figure 3E: The whole panel E is labelled “Macrophages PCs Overlay” and this is misleading. Each individual panel of Fig3E should be labeled either”transmission”(upper left) “macrophages”(lower right) or ‘PCs”(upper right) or “overlay”(lower left) while “11hpfr/3hpi” should apply to the wole panel 3E.

Done.
Figure 3G & 3H: Same as for 3E, please correctly label each individual panel.
Done.

Figure 3J-N : Text and numbers are unreadable, please use larger fonts for all text and all numbers even for the axis numbering.
Done.

Line 202: we live-stained Tg(osx:mCherry) adult fish (with RFP-tagged osteoblasts). Should be “we live-stained Tg(osx:mCherry) adult fish (with mCherry-tagged osteoblasts).”

Thanks for this; we have reworded as you suggested.
Lines 206-209: The authors should refer to the TRAP labelling of osteoclasts.
Yes, TRAP staining now described, p9.

© 2024. Published by The Company of Biologists under the terms of the Creative Commons Attribution License (https://creativecommons.org/licenses/by/4.0/).

FigS4D: Please write “systemic infection” similar to what is written in S4A and S4H.
Done.

Fig S4I: Similar critic as for Fig1I and Fig3E.
Done.

FigS4I: “48hpinf” applies to the whole panel or just to the upper left image?
It applies to whole panel and so we have moved it as suggested.

Fig 4: All texts are un readable, please use a larger font size.
As mentioned earlier. Now we have split these figures which has given us more space for increased font size.

Fig4B: the IL1b panel highlight the fact that a linear scale is not appropriate to display value ranging more than on log. It is necessary in such a case to use a log scale. Please modify!
Done.

Fig 4C: Same as for 4B, it is necessary to use a log scale for all panels… except (perhaps) for IL1RA.
Done.

Fig 4D: “40mpinf”” refers to all 4 images, please write “40mpinf” nest to E.coli.
Done.

Line 274: “resuspended in 2% PVP40 solution (Sigma) and phenol red (Sigma).” Please explain what exactly you mean by “2% PVP40 solution (Sigma)”? … is it buffered? … and why you use PVP.
We now explain how we buffered PVP and why we used this solution for our experiments in Methods, p13.

Line 327: “Larvae were anaesthetized at 48 hpf in MS-222 (Sigma)”. MS-222 is a commercial reference, not a product, I suggest that the authors write ““Larvae were anaesthetized at 48 hpf using tricaine {please indicate the final concentration} (Sigma MS-222)”
Done.

Line 331, 340, 346, 366, 371: Same as for line 327.
Done.

Line 347: “blunt-ended glass capillary tube”. Reference and/or details?
Reference and source company details have been included.

Line 353: “pH adjusted to 8”… using what?
Done.

Lines 481-483: It is not normal that datasets availability would depend on a person. They should be available using DOIs on public/permanent repositories.
All datasets from this study are now available at Zenodo repository and the link to access these is provided in the “Data availability statement” section.

*****

Reviewer 3 Advance Summary and Potential Significance to Field:
In this work, López-Cuevas et al. present compelling data on the use of artificial protocells and a TLR7/8 agonist to modulate wound healing in two zebrafish wounding models with some extension to human macrophages. This work follows up a 2022 paper from the same group in which an anti-miR223 cargo is delivered in the context of an in vivo tumor model. There are nicely characterized effects on macrophage profile and wound healing processes, expertly characterized throughout the paper, and, finally, the authors demonstrate an effect on bacterial phagocytosis of human peripheral blood- derived macrophages. The breadth of assays used (and careful characterization therein) suggests that this strategy may have general utility, and the retroorbital injections in adults followed by bone fracture were particularly impressive. Overall there are two major additions that would considerably strengthen the manuscript.

© 2024. Published by The Company of Biologists under the terms of the Creative Commons Attribution License (https://creativecommons.org/licenses/by/4.0/).

Reviewer 3 Comments for the Author:
1) The protocells are a very nice and novel approach, but I couldn't find any direct comparison to just administering the same drug in similar quantities, either through injection or even immersion. This would be very helpful to formally demonstrate potential advantages of the protocells.
We have performed the experiment to directly compare R848-protocells versus free R848 and showed that R848-protocells delivery triggered more macrophage reprogramming, characterised by an increase in the number of tnfa-positive macrophages, indicating that this protocol is more efficacious than free R848 administration. These data are in Fig. S2 and described in the Results section (lines 162, 166, 167).

2) The assumption is that the drug acts as a TLR7/8 agonist (and that these molecules might normally be inaccessible to the drug). This should really be tested for specificity in some way other than pro-inflammatory output. I recognize that there are multiple TLR7/8 paralogues in zebrafish so a comprehensive genetic approach would be difficult, but there should be some way to build evidence for or against, particularly as I assume that this target has not been demonstrated before in zebrafish. Multiplexed CRISPR with assessment of crispants? Perhaps an antagonist? Or perhaps interfering with TLR7/8 via something like the unc93 pathway? Since most of the paper focuses on zebrafish, I think it is important to at least attempt to show some specificity there, but lower priority conceptually could go to the human system and inhibition there (siRNAs?).
We understand that showing the specificity of R848 for TLR7/8 in zebrafish would strengthen our manuscript, and we have added a line as part of our Discussion section (lines 357, 358) to flag this up. However, we believe this experimental demonstration is outside the scope of our study. There is good evidence from the literature (Bottiglione et al., 2020; Jault et al., 2004; Meijer et al., 2004; Progatzky et al., 2019) that the sequence and function of TLR7/8 are very well conserved between zebrafish and mammals, suggesting that the interactions of R848 with TLR7/8, which have been shown to be specific in mammals (Hemmi et al., 2002; Jurk et al., 2002), should also occur in zebrafish. In addition, R848 drug has been used to drive a pro- inflammatory response in the zebrafish model in multiple studies (Bottiglione et al., 2020; Muire et al., 2017; Progatzky et al., 2019; Ward and Martin, 2023). We have spelled out these arguments (+ citation support) on p3 and p11.

The introduction is short, as is the discussion. All fine, particularly for a short report format, but I do think there is an opportunity to orient the reader a bit more about protocells (advantages, delivery, etc) as well as more robust discussion of how strongly the TLR7/8 activation should be pursued as a therapeutic strategy with pitfalls or whether this manuscript represents further proof of principle in the context of infected wounds. For the therapeutic discussion generally, may be worth commenting on the pre-administration that is used in the manuscript to see full effect. And since many of the assays show impairments with protocells, it might be nice to have at least some of Figure S4 (enhanced uptake and killing of bacteria upon PC treatment) as part of a main figure or a standalone figure.
As mentioned in response to Ref 1's concerns, we have expanded the Intro and Discussion. Advantages of protocells over other delivery methods (eg. liposomes) are clearly spelled out in the last paragraph of the Intro (p3,4) and in the second paragraph of Discussion (p10,11).
We also comment on the potential future clinical applications of protocells in the last paragraph of Discussion (p12). There is no mention of the pre-administration aspect of protocells because this may not be directly translated into the clinic. In our zebrafish experiments, we administered two protocell doses, pre- and post-wounding, because fish could not tolerate higher protocell concentration in a single injection, but this might be feasible when upscaling into the human situation. Finally, we have moved some of the data related to enhanced killing of bacteria upon PC treatment from a previous Suppl. Fig. so that they are now in Fig. 4B,E.

Finally, just a general compliment about the quality of the imaging and the movies!
Thanks for this; much appreciated.

We hope that our additional data as well as our responses to your reviewers' concerns, above, mean that you now feel that our paper is suitable for publication as an article in JCS.

Best regards

Paul Martin and co-authors

© 2024. Published by The Company of Biologists under the terms of the Creative Commons Attribution License (https://creativecommons.org/licenses/by/4.0/).

Second decision letter

MS ID#: JOCES/2024/262202

MS TITLE: Reprogramming macrophages with R848-loaded artificial protocells to modulate skin and skeletal wound healing

AUTHORS: Paco Lopez-Cuevas, Tiah CL Oates, Qiao Tong, Lucy M McGowan, Stephen J Cross, Can Xu, Yu Zhao, Zhuping Yin, Ashley M Toye, Asme Boussahel, Chrissy L Hammond, Stephen Mann, and Paul Martin

I am happy to tell you that your manuscript has been accepted for publication in Journal of Cell Science, pending standard publication integrity checks.

Reviewer 1

Advance summary and potential significance to field

The authors have addressed all my comments.

Comments for the author

The authors have addressed all my comments.

Reviewer 2

Advance summary and potential significance to field

The manucript « Reprogramming macrophages with R848-loaded artificial protocells to modulate zebrafish skin and skeletal wound healing" by Paco Lopez-Cuevas, Tiah CL Oates, Qiao Tong, Lucy M McGowan, Stephen J Cross, Can Xu, Yu Zhao, Zhuping Yin, Ashley M Toye, Asme Boussahel, Chrissy L Hammond, Stephen Mann and Paul Martin reports the reprogramming of zebrafish macrophages toward a proinflammatory and bactericidal phenotype using R848 loaded protocells. Their results suggest that human macrophages do behave the same. They also show that this proinflammatory reprogramming of macrophages partially impairs wound healing.

Comments for the author

All comments from the reviewers have been appropriately addressed.

Reviewer 3

Advance summary and potential significance to field

See previous review.

Comments for the author

The authors have done a very nice job with the revisions. Although they were not able to provide genetic evidence of specificity, they make a good case about why this is beyond the scope of the work as well as being particularly challenging in zebrafish, and they have provided additional controls showing the effects of the protocells.

© 2024. Published by The Company of Biologists under the terms of the Creative Commons Attribution License (https://creativecommons.org/licenses/by/4.0/).
